# NO at low concentration can enhance the formation of highly oxygenated biogenic molecules in the atmosphere

**DOI:** 10.1038/s41467-023-39066-4

**Published:** 2023-06-08

**Authors:** Wei Nie, Chao Yan, Liwen Yang, Pontus Roldin, Yuliang Liu, Alexander L. Vogel, Ugo Molteni, Dominik Stolzenburg, Henning Finkenzeller, Antonio Amorim, Federico Bianchi, Joachim Curtius, Lubna Dada, Danielle C. Draper, Jonathan Duplissy, Armin Hansel, Xu-Cheng He, Victoria Hofbauer, Tuija Jokinen, Changhyuk Kim, Katrianne Lehtipalo, Leonid Nichman, Roy L. Mauldin, Vladimir Makhmutov, Bernhard Mentler, Andrea Mizelli-Ojdanic, Tuukka Petäjä, Lauriane L. J. Quéléver, Simon Schallhart, Mario Simon, Christian Tauber, António Tomé, Rainer Volkamer, Andrea C. Wagner, Robert Wagner, Mingyi Wang, Penglin Ye, Haiyan Li, Wei Huang, Ximeng Qi, Sijia Lou, Tengyu Liu, Xuguang Chi, Josef Dommen, Urs Baltensperger, Imad El Haddad, Jasper Kirkby, Douglas Worsnop, Markku Kulmala, Neil M. Donahue, Mikael Ehn, Aijun Ding

**Affiliations:** 1 Joint International Research Laboratory of Atmospheric and Earth System Research, School of Atmospheric Sciences, Nanjing University, Nanjing, China; 2 National Observation and Research Station for Atmospheric Processes and Environmental Change in Yangtze River Delta, Nanjing, Jiangsu Province China; 3 Institute for Atmospheric and Earth System Research/Physics, Faculty of Science, University of Helsinki, Helsinki, Finland; 4 Department of Physics, Lund University, P. O. Box 118, SE-221 00 Lund, Sweden; 5 IVL, Swedish Environmental Research Institute, SE-211 19 Malmö, Sweden; 6 Institute for Atmospheric and Environmental Sciences, Goethe University Frankfurt, Frankfurt am Main, 60438 Germany; 7 Laboratory of Atmospheric Chemistry, Paul Scherrer Institute, 5232 Villigen PSI, Switzerland; 8 Department of Chemistry, University of California, Irvine, CA 92697 USA; 9 Forest Dynamics, Swiss Federal Institute for Forest, Snow and Landscape Research, 8903 Birmensdorf, Switzerland; 10 Faculty of Physics, University of Vienna, Boltzmanngasse 5, 1090 Vienna, Austria; 11 Department of Chemistry & CIRES, University of Colorado Boulder, Boulder, CO 80309 USA; 12 CENTRA and FCUL, Universidade de Lisboa, Campo Grande, 1749-016 Lisboa Portugal; 13 Helsinki Institute of Physics (HIP)/Physics, Faculty of Science, University of Helsinki, 00014 Helsinki, Finland; 14 Institute of Ion and Applied Physics, University of Innsbruck, 6020 Innsbruck, Austria; 15 Center for Atmospheric Particle Studies, Carnegie Mellon University, Pittsburgh, PA USA; 16 Climate & Atmosphere Research Centre (CARE-C), The Cyprus Institute, P.O. Box 27456, Nicosia, CY-1645 Cyprus; 17 School of Civil and Environmental Engineering, Pusan National University, Busan, 46241 Republic of Korea; 18 Division of Chemistry and Chemical Engineering, California Institute of Technology, Pasadena, CA 91125 USA; 19 Finnish Meteorological Institute, Erik Palménin aukio 1, 00560 Helsinki, Finland; 20 Flight Research Laboratory, National Research Council Canada, Ottawa, K1A 0R6 ON Canada; 21 Department of Atmospheric and Oceanic Sciences, University of Colorado Boulder, Boulder, CO USA; 22 P.N. Lebedev Physical Institute of the Russian Academy of Sciences, 53, Leninskiy Prospekt, Moscow, Russian Federation; 23 Moscow Institute of Physics and Technology (National Research University), 1A Kerchenskaya st., Moscow, Russian Federation; 24 Ion Molecule Reactions & Environmental Physics Group Institute of Ion Physics and Applied Physics Leopold-Franzens University, Innsbruck Technikerstraße 25, A-6020 Innsbruck, Austria; 25 Faculty of Industrial Engineering, FH Technikum Wien - University of Applied Sciences, 1200 Vienna, Austria; 26 IDL-Universidade da Beira Interior, Rua Marquês D’Ávila e, Bolama, 6201-001 Covilhã Portugal; 27 Shanghai Key Laboratory of Atmospheric Particle Pollution and Prevention (LAP3), Department of Environmental Science and Engineering, Fudan University, Shanghai, 200438 China; 28 School of Civil and Environmental Engineering, Harbin Institute of Technology, Shenzhen, 518055 China; 29 CERN, CH-1211 Geneva, Switzerland; 30 Aerodyne Research Inc., Billerica, MA 01821 USA; 31 Present Address: Division of Geological and Planetary Sciences, California Institute of Technology, Pasadena, CA 91125 USA; 32 Present Address: Department of Chemistry & CIRES, University of Colorado Boulder, Boulder, CO 80309 USA

**Keywords:** Atmospheric chemistry, Geochemistry

## Abstract

The interaction between nitrogen monoxide (NO) and organic peroxy radicals (RO_2_) greatly impacts the formation of highly oxygenated organic molecules (HOM), the key precursors of secondary organic aerosols. It has been thought that HOM production can be significantly suppressed by NO even at low concentrations. Here, we perform dedicated experiments focusing on HOM formation from monoterpenes at low NO concentrations (0 – 82 pptv). We demonstrate that such low NO can enhance HOM production by modulating the RO_2_ loss and favoring the formation of alkoxy radicals that can continue to autoxidize through isomerization. These insights suggest that HOM yields from typical boreal forest emissions can vary between 2.5%-6.5%, and HOM formation will not be completely inhibited even at high NO concentrations. Our findings challenge the notion that NO monotonically reduces HOM yields by extending the knowledge of RO_2_-NO interactions to the low-NO regime. This represents a major advance towards an accurate assessment of HOM budgets, especially in low-NO environments, which prevails in the pre-industrial atmosphere, pristine areas, and the upper boundary layer.

## Abstract (web summary)

Nitrogen monoxide at low concentrations can promote the formation of highly oxygenated organic molecules, which are crucial precursors to secondary organic aerosol (SOA), rather than inhibit it as traditionally thought.

## Introduction

Atmospheric aerosols are crucial to Earth’s radiative forcing and climate, yet their influence remains poorly quantified^1^. Globally, organic aerosols contribute significantly to the total aerosol mass^2–5^. Oxidation pathways of various volatile organic compounds (VOCs) involves autoxidation of peroxy (RO_2_) radicals^6^. The products, known as highly oxygenated organic molecules (HOM)^7^, have recently been observed in various environments in the atmosphere. Owing to their high oxidation state and low volatility, HOM are a major source of secondary organic aerosol (SOA)^8^ and contribute significantly to new particle formation (NPF)^9–11^.

NO_x_ concentrations are substantially elevated in the present-day atmosphere due to anthropogenic emissions. NO_x_ profoundly influences HOM formation. First, it can regulate the atmospheric oxidation capacity and consequently affect the oxidation of VOCs^12–15^; Second, NO_x_ can greatly influence the extent of RO_2_ radical autoxidation^16^ and thus HOM composition by directly reacting with RO_2_ radicals, leading to enhanced formation of organic nitrates and suppressed formation of HOM dimers^6,17–25^. Up to now, the second effect remains poorly quantified, presenting an important obstacle towards a complete understanding of HOM budget and its impacts on aerosol formation in the atmosphere. Currently, most laboratory experiments have been conducted either at effectively zero NO_x_ (with very high VOC: NO_x_), or at relatively high NO_x_ concentrations. It is widely accepted that high NO_x_ suppresses RO_2_ autoxidation and HOM formation^16^. Yet, little is known about HOM formation at low but non-zero NO_x_ concentrations (i.e., NO ranges from 5–100 pptv and NO_2_ ranges from 0.1–5 ppbv), characteristic of the pre-industrial atmosphere, the pristine environments, the upper boundary layer, and likely the future atmosphere if NO_x_ concentration continuously declines. Because the interactions between RO_2_ and NO_x_ are so complex (involving the different roles of NO, NO_2_, and NO_3_), the overall influence on HOM formation can be highly nonlinear. Therefore, simply extrapolating the results derived from high-concentration experiments to low-concentration conditions, or even interpolating between high-NO_x_ and effectively zero NO_x_, may lead to substantial biases^26,27^.

In this work, we perform dedicated experiments at the CERN CLOUD (Cosmics Leaving Outdoor Droplets) chamber with molecular-level observations and simulations focusing on the HOM formation at low NO concentrations^28^. We demonstrate that the role of NO_x_ in HOM formation is more nuanced than simple suppression, and low NO concentrations can cause a previously overlooked increase in HOM yields.

## Results and discussion

### An overview of experimental observations

**Fig. 1:**
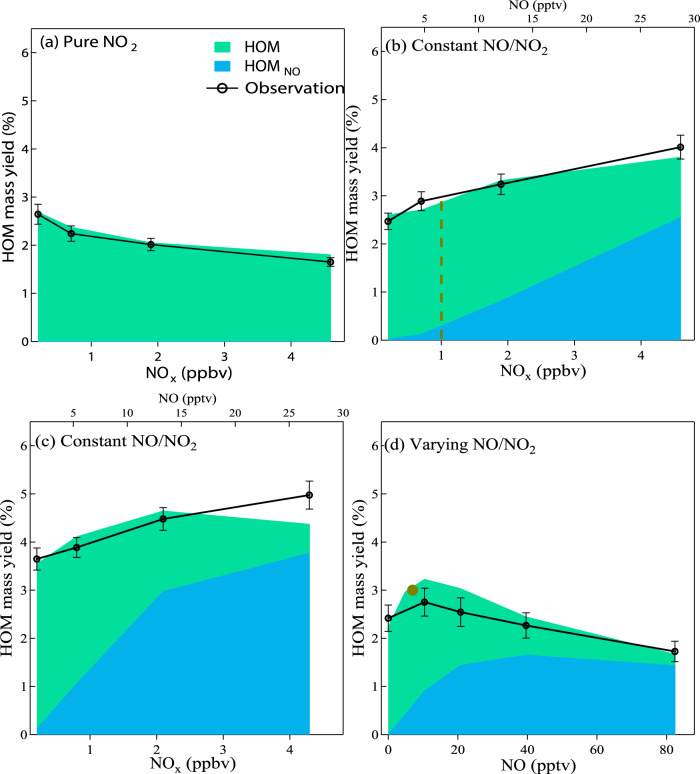
Comparison of measured and modelled mass yields of highly oxygenated organic molecules (HOM). In (**a**) a pure NO_2_ experiment run with 1200 pptv monoterpene, (**b**) a constant NO/NO_2_ experiment with 1200 pptv monoterpene, (**c**) a constant NO/NO_2_ experiment with 300 pptv monoterpene and (**d**) a varying NO/NO_2_ experiment with around 1 ppbv NO_2_ and 1200 pptv monoterpene. The brown solid circle in (**d**) denotes the HOM mass yield obtained from the constant NO/NO_2_ experiment with around 1 ppbv NO_2_ and 7 pptv NO (the brown dash line in (**b**)). Model simulated HOM represents molecules formed without NO participation and are denoted in green; HOM_NO_ represents molecules formed with NO’s involvement and are denoted in blue. The propagated error of the HOM yield varies within 6–8% among different experiments with the calculation method provided in the Methods.

In the experiments, we kept the chamber conditions stable at a temperature of 278 K and 38% relative humidity. A 2:1 mixture of α-pinene and Δ−3-carene comprised the VOC precursors, representative of the typical monoterpene profile above the canopy in a boreal forest in southern Finland (the SMEAR II station, the reference site for this study)^29,30^. In different experiments, the steady-state mixing ratio of total monoterpenes was approximately 300, 600, and 1200 pptv. We controlled NO_x_ in three distinct sequences: experiments with only NO_2_ to represent the nighttime condition (Fig. 1a); experiments with variable NO_x_ concentrations with a constant NO to NO_2_ ratio (~0.007) to represent the morning (Fig. 1b, c); and experiments at constant NO_x_ with variable NO to NO_2_ ratios to represent the evolution from night to noon (Fig. 1d). These experiments are referred to as “pure NO_2_”, “constant NO/NO_2_”, and “variable NO/NO_2_” hereafter in this work. The highest NO_2_ and NO mixing ratios in these experiments were 4.6 ppbv and 82 pptv, respectively. A vital feature of these experiments is the precise control and monitoring of NO and monoterpenes at low concentrations, which to the best of our knowledge, has never been achieved in previous studies. This allows us to successfully reproduce the chemical environment of the boreal forest atmosphere, and to investigate the HOM production in detail in the low-NO regime. A list of experiments illustrated in Fig. 1 is provided in Supplementary Table 1, and detailed information on the experimental design and instrumentation is provided in Methods.

A nitrate-based chemical ionization mass spectrometer (CI-APi-TOF)^6,31^ was deployed to measure gas-phase HOM; these were classified as molecules without (CHO) and with nitrogen atoms (CHON), the latter likely being organic nitrates. We show the variations of some fingerprint CHON- and CHO- HOM for different monoterpene and NO_x_ values in Supplementary Fig. 1. The corresponding dependence of HOM concentrations and mass yields on NO_x_ is illustrated in Fig. 1 and Supplementary Fig. 2a–d. The response of HOM production to NO_x_ levels shows marked differences for different NO_x_ sequences, indicating that NO_x_ influences HOM formation in a complicated manner. For example, in the pure NO_2_ sequence, the total HOM yield decreases monotonically as the NO_2_ concentration rises (Fig. 1a). However, in a nearly identical sequence with constant NO/NO_2_, differing only by a small amount of NO (maximum 28 pptv), HOM yields increase monotonically, from about 2.5% to 4.0% as the NO_x_ concentration rises (Fig. 1b). This enhancement is more pronounced at low monoterpene concentrations (Fig. 1c) than at high monoterpene concentrations when the NO_x_ concentration is identical. These results clearly show that the roles of NO and NO_2_ in HOM formation are different – while NO_2_ suppresses HOM formation monotonically^32^, NO at low concentrations can enhance HOM formation. The influence of NO on HOM yields is seen more clearly in the varying NO/NO_2_ sequence; When NO increases from 0 to 82 pptv at an approximately constant NO_2_ concentration near 1 ppbv, it first enhances HOM production, but as it further increases the enhancement gradually diminishes and turns into suppression (Fig. 1d). Altogether, these observations clearly show that NO has a non-linear effect on HOM yields, challenging the notion that NO monotonically suppresses HOM production by inhibiting the autoxidation of RO_2_^15,16^.

### A mechanistic understanding of the roles of NO_x_ in HOM formation

We use the Aerosol Dynamics gas- and particle-phase chemistry model for laboratory CHAMber studies (ADCHAM) to further obtain a mechanistic understanding of the influence of NO on HOM formation^11^. This model simulates HOM formation on a molecular level. We extend the peroxy radical autoxidation mechanism (PRAM) module^11,33^ in ADCHAM by incorporating NO_3_ chemistry and refining the representation of alkoxy radical (RO) isomerization, which better describes HOM formation through the interactions between monoterpenes and NO_x_. As shown in Fig. 1 and Supplementary Fig. 2, the model not only reproduces the HOM distribution at various experimental conditions, but also captures the variation of HOM concentrations for the different NO_x_ sequences we employed in our experiments, showing that the model setup is reasonable and results are robust.

With the help of ADCHAM, we track each reaction channel and divide HOM into HOM_NO_ and HOM_non-NO_. HOM_NO_ denotes HOM formation directly involving NO in at least one reaction, forming either an organic nitrate or an RO radical. HOM_non-NO_ denotes HOM formation without any reactions involving NO. We can see that the HOM_NO_ increase significantly in three NO-involved sequences (Fig. 1b-d). The fraction of HOM_NO_ is approximately 85 % at low monoterpene concentrations (Fig. 1c) or high NO (Fig. 1d). This high fraction of HOM_NO_ in the model also is consistent with our PMF analysis (see Methods and Supplementary Fig. 3). This also agrees with our previous PMF analysis of ambient data at our reference site, where the dominant daytime factors are NO-involved (accounting for ~90% of the total HOM concentration) with around 80 pptv NO (Supplementary Fig. 4)^17^, suggesting NO is involved in the formation of the majority of HOM at quite low concentrations.

Next, we use the varying NO/NO_2_ sequence to investigate the non-linear effects of NO on HOM yields. We find two main causes. First, NO suppresses RO_2_ production and slows the second-order RO_2_ cross-reactions dramatically. When these cross-reactions are the dominant RO_2_ sink, they can terminate RO_2_ chemistry before isomerization reactions initiate autoxidation. Second, NO enhances RO formation, which also undergoes autoxidation and leads to second-generation RO_2_. Both these effects enhance HOM formation when RO_2_ + NO is not the major RO_2_ sink but suppress HOM formation when RO_2_ + NO becomes the dominant RO_2_ sink. This drives the nonlinear HOM production with a peak at low but non-zero NO.

**Fig. 2:**
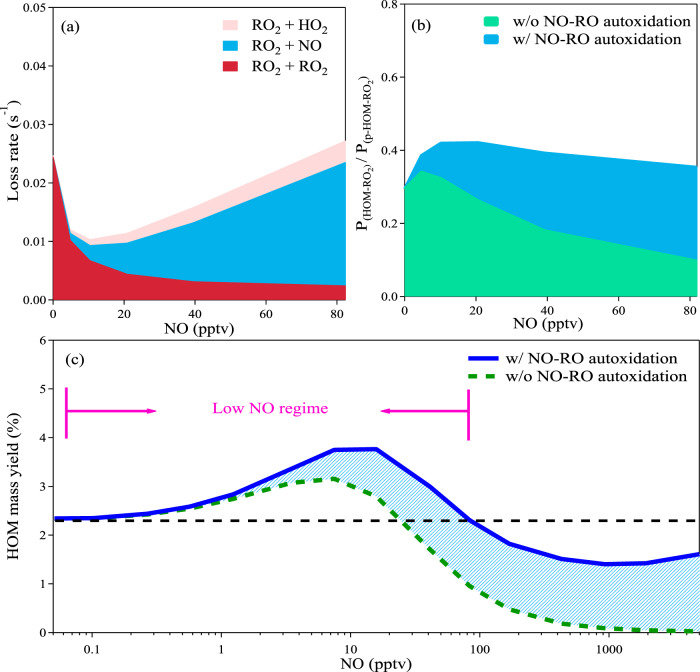
The mechanism through which NO can enhance the formation of highly oxygenated organic molecules (HOM) from monoterpene oxidation at 278 K. (**a**) Loss rates of p-HOM-RO_2_ (RO_2_ that can undergo autoxidation with *n*_O_ < 7) as a function of NO concentration in the varying NO/NO_2_ experiment. Reactions of RO_2_ + HO_2_, RO_2_ + NO, and RO_2_ + RO_2_ are marked by pink, blue, and red, respectively. (**b**) The fraction of p-HOM-RO_2_ that can undergo autoxidation to HOM-RO_2_ with (the sum the blue and green fillings) and without (green fillings) NO-induced RO autoxidation in the varying NO/NO_2_ experiment. (**c**) Comparison between HOM yields with (solid blue line) and without NO (dashed green line) RO autoxidation. In the simulation of Fig. 2c, the concentrations of monoterpene, O_3_, and NO_2_ are constrained at 1200 pptv, 40 ppbv and 1 ppbv.

An important detail is that only some RO_2_ can undergo rapid autoxidation. According to the latest understanding of bicyclic monoterpene oxidation, only a fraction of first-generation RO_2_ can undergo cyclobutyl ring breaking, a necessary step for further autoxidation and formation of HOM-RO_2_^34^. This suggests a fixed yield of RO_2_ that can undergo autoxidation. The non-linear effect of NO on HOM yield is essentially caused by influencing the further oxidation of these potential HOM-RO_2_ (p-HOM-RO_2_, defined as RO_2_ that can undergo autoxidation with *n*_O_ < 7) to HOM-RO_2_ (defined as RO_2_ that is autoxidized to *n*_O_ ≥ 7, Fig. 2b), e.g., from C_10_H_15_O_4_ to C_10_H_15_O_≥7_ in the ozonolysis of monoterpene. In Fig. 2a, we show the loss rate of the p-HOM-RO_2_ at different NO levels. NO has a non-linear influence on the loss rate of the p-HOM-RO_2_ and thus the possibility that p-HOM-RO_2_ becomes HOM-RO_2_. When NO is absent, p-HOM-RO_2_ loss is primarily due to the cross-reaction with non-HOM-RO_2_ (defined as RO_2_ that cannot autoxidize). Low but non-zero NO (e.g., 10 pptv) drastically reduces the total loss rate of p-HOM-RO_2_ by efficiently reducing the concentration of non-HOM-RO_2_. Although NO also consumes p-HOM-RO_2_, at low NO this effect is more than compensated by the decrease in non-HOM-RO_2_, which dominates the loss of p-HOM-RO_2_ at this NO level. As the NO concentration further increases, it progressively becomes the dominant sink of the p-HOM-RO_2_ (Fig. 2a) and starts to inhibit HOM production. This intervention in the RO_2_ cross-reactions can also partially explain the suppression effect of NO_2_ on HOM formation, in addition to the termination of chain propagation of acyl RO_2_^31^. Elevated NO_2_ produces considerable non-HOM-RO_2_ via NO_3_ oxidation, which reduces the probability of p-HOM-RO_2_ producing HOM.

Aside from modulating the HOM-RO_2_ loss rate, NO also affects the formation of HOM-RO_2_ directly by converting RO_2_ to RO. As studied under high-NO conditions^35^, a fraction of RO can isomerize and form new RO_2_ via intra-molecular hydrogen abstraction followed by instantaneous O_2_ addition. In the context of HOM formation, we refer to RO isomerization as the “RO autoxidation channel” (Supplementary Fig. 5), as it is similar to the definition of RO_2_ autoxidation. It should be noted that the RO autoxidation channel has been shown to play a non-negligible role in HOM formation following α-pinene ozonolysis under NO_x_-free conditions, as RO_2_ cross-reactions also lead to RO formation^36^. But the presence of NO leads to additional RO formation, making this channel more important. In the case of alkane photo-oxidation, which can have very low yields of highly oxygenated products, the addition of NO was recently shown to greatly enhance the concentration of the most oxygenated products^37^. This was inferred to be due to several steps of RO isomerization taking place, causing the efficient formation of highly oxygenated species even at 10 ppbv NO.

We cannot directly measure the short-lived RO radicals, so we use distinct HOM products as markers to examine the importance of this channel. In the ozonolysis of monoterpenes, HOM-RO_2_ produced via direct autoxidation contain an odd number of hydrogen atoms (*n*_H_) and an even number of oxygen atoms (*n*_O_), e.g., C_10_H_15_O_8,10_. The corresponding closed-shell HOM from either unimolecular termination or bimolecular reactions with RO_2_ and HO_2_ must be C_10_H_14,16_O_7,9_ and C_10_H_16_O_8,10_ (Supplementary Fig. 5), which are the major HOM products in many experiments without NO_x_^6,36,38^. In contrast, RO isomerization will produce an RO_2_ with an odd *n*_O_, and the consequent closed-shell species are e.g. C_10_H_14_O_8,10_ (Supplementary Fig. 5). In Supplementary Fig. 6, we show the variation of C_10_H_14_O_8,10_ at different NO levels. As NO rises, they increase by *ca*. 30 and 100 %, respectively, illustrating the enhancement of the RO autoxidation channel (Fig. 2b). In Fig. 2c, we show the importance of RO autoxidation by comparing the results with and without this reaction channel. The ADCHAM model can only reproduce the experimental data when the RO reaction channel is implemented. Without the RO reaction channel, the predicted HOM yield is significantly lower than the experimental observation, and the difference diverges at higher NO concentrations. This channel forms *ca*. 25% of HOM even at only 10 pptv NO, and it is responsible for almost all the HOM formation at above 100 pptv NO. Because of this, the HOM yield remains at a considerable level at high NO concentration, although still lower than the yield under NO-free conditions. It is worth noting that RO autoxidation also explains the large fraction of CHO-HOM formed with the direct involvement of NO (Supplementary Fig. 2).

**Fig. 3:**
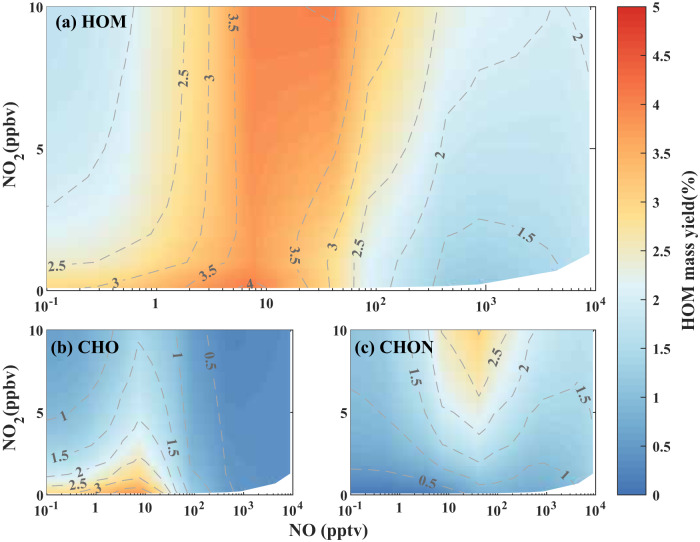
Model simulated non-linear dependence of the yields of highly oxygenated organic molecules (HOM) on NO_2_ and NO at 278 K. (**a**) total HOM, (**b**) CHO-HOM, and (**c**) CHON-HOM with 1200 pptv monoterpene. O_3_ concentrations are constrained at 40 ppbv at each experiment. The contour lines indicate the corresponding HOM yields.

We summarize the dependences of HOM yields on NO and NO_2_ in Fig. 3. In general, NO_2_ suppresses HOM production monotonically, and NO has a non-linear effect (Fig. 3a). It was previously thought that HOM suppression by NO_2_ was mostly due to the direct reaction of RO_2_ + NO_2_ outcompeting RO_2_ autoxidation^32^. However, we find that NO_2_ can suppress HOM production indirectly by increasing the loss rate of HOM-RO_2_ - it promotes NO_3_-initiated monoterpene oxidation, which forms a considerable amount of non-HOM-RO_2_, enhancing the second-order RO_2_ cross-reactions. When NO is also present, it counteracts the suppression effects of NO_2_: on the one hand, it can significantly consume NO_3_; on the other hand, it reduces the total loss rate of HOM-RO_2_ (Fig. 2a). Our results suggest that the highest HOM yield at different NO_2_ levels is similar when NO is present, although the optimal NO concentration varies slightly. This makes the enhancement of HOM yield by NO more significant at high NO_2_ (up to 140% with NO_2_ at 10 ppbv, Fig. 3a). The enhancement of HOM yield at low NO is pronounced for CHO HOM at low NO_2_ concentrations (e.g., <5 ppbv) (Fig. 3b) and for CHON HOM at high NO_2_ concentrations (e.g., >5 ppbv) (Fig. 3c).

It is worth noting that the quantitative relationships outlined here may be sensitive to conditions in human-influenced environments, where a greater abundance of non-HOM-RO_2_ originating from small molecule VOCs may lead to a stronger loss of HOM-RO_2_. In this case the maximum HOM yield is expected to occur at a higher concentration of NO.

### Atmospheric observational evidence and implications

**Fig. 4:**
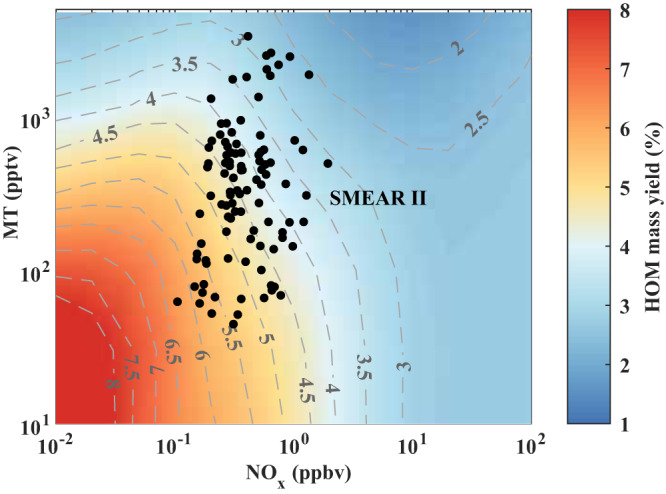
Contour plot of the yields of highly oxygenated organic molecules (HOM) from monoterpene oxidation vs monoterpene and NO_x_ concentration. The observation data of monoterpene and NO_x_ from the SMEAR II boreal forest station^58^ are used to predict the HOM yield (black points). A series of simulations were conducted with monoterpene concentration varying from 100 to 1800 pptv, and NO_x_ concentration from 0.01 to 100 ppbv. With the assumption NO is solely formed from NO_2_ photolysis, NO/NO_2_ varied to follow the observed diurnal cycle at SMEAR II station from almost zero at night to 14.3% at 10 am. We take the daytime average HOM yield as the HOM yield at fixed NO_x_ and monoterpene concentration.

We demonstrate that HOM formation is governed by a complex competition between multiple reactions including RO_2_ autoxidation, RO isomerization, and various uni-molecular and bi-molecular termination reactions. Therefore, HOM yields vary depending on NO, NO_2_, and monoterpene concentrations and are expected to vary significantly in different environments rather than remain constant. As shown in Fig. 4, we reproduce the HOM yields in a boreal forest in southern Finland by implementing these insights. The details of the simulation inputs and assumptions are provided in Methods. The calculated HOM yield as a function of measured NO_x_ and monoterpene concentrations varies by almost a factor of three, from 2.5% to 6.5%. This large variation can cause a notable change in both SOA formation and particle growth.

Although this study investigates HOM formation at low and moderate NO levels, the highlighted importance of the RO autoxidation channel also advances our understanding of HOM formation at high NO concentrations, common for monoterpene oxidation in the polluted urban boundary layer. It has been argued that HOM formation can be completely suppressed when the RO_2_ + NO reaction overwhelms RO_2_ autoxidation^15^. However, our measurements in polluted East China show that the HOM yield of monoterpene oxidation remains at approximately 1–2% on average (Supplementary Fig. 7), instead of approaching zero as anticipated. This discrepancy can be explained by RO autoxidation, as we demonstrated in Fig. 2c. Besides monoterpene oxidation, the RO autoxidation channel should be important in the oxidation of other VOCs, such as alkanes^8,37^. This is consistent with the significant anthropogenic HOM production observed in polluted megacities with high NO concentrations^8^. We conclude that high NO cannot completely inhibit HOM formation even in severely polluted urban environments.

In summary, combining experimental data, ambient observations, and molecule-level model simulations, we demonstrate that NO has a non-linear effect on HOM formation. Though NO_x_ maxima are known for phenomena such as O_3_ production and HO_x_ concentrations, the maxima occur at different points. For HOM production, the maximum is at very low NO, meaning that environments can be “low NO_x_” for O_3_ yet “high NO_x_” for HOM. We propose a new division of NO regimes including “Zero NO_x_”, “Low NO”, and “High NO” (Supplementary Table 2) in HOM-related studies. In this new division, previous studies almost completely overlooked the low-NO regime, in which HOM formation is enhanced due to a reduced sink of HOM-RO_2_ as well as enhanced RO autoxidation. Conceptually, this non-linear effect is similar to the radical termination via production of organonitrates (at intermediate NO_x_)^39^ and even the catalytic ozone depletion in the stratosphere (with NO_x_ suppressing HO_x_ and ClO_x_ catalysis at low concentrations but ultimately enhancing catalytic ozone loss at sufficiently high NO_x_)^40^. All these reveal the notably different outcomes between low-concentration and high-concentration reactions in atmospheric photochemistry. In fact, from a global perspective, high-NO environments are the exceptions, existing only near the ground surface of regions with strong human activity, whereas low-NO environments likely prevail in the vast majority of Earth’s boundary layer. As shown in Supplementary Fig. 8, the low-NO regime (NO < 30 pptv) prevailed at our boreal-forest reference site, accounting for more than half of the observation period from 2002 to 2018. In a megacity of east China, although only 6% of the observation data was lower than 30 pptv, the frequency of low-NO conditions has increased significantly in recent years due to NO_x_ emission controls. Therefore, this refined understanding of HOM formation in the low-NO regime is crucial for an accurate evaluation of HOM and aerosol budgets, as well as climate change.

## Methods

### The CLOUD chamber

The CLOUD chamber is a well-controlled stainless-steel cylinder with a volume of ca. 26.1 m^3^, located at CERN, Geneva, Switzerland^28,41^. Plenty of efforts were made to keep the chamber ultra-clean, including using an electro-polished inner surface, rinsing with 373 K ultrapure water and flushing the chamber with humidified synthetic air containing several ppmv of ozone thereafter before each campaign. Synthetic air used throughout the campaign was produced by mixing ultra-clean cryogenic liquid nitrogen and oxygen. These efforts achieved an as low as possible contamination level that the background VOCs concentration is below ppbv level and the condensable vapors are mostly below the detection limits, and allowed investigating the chemical processes with atmospherically relevant concentration of reaction precursors^42^, e.g. pptv level monoterpene and NO_x_. It is worth noting that with the ultra-clean electro-polished stainless-steel surface, the influence of HO_2_ produced from the chamber wall which was a long-standing trouble for Teflon chambers is believed to be ignorable during the campaign.

Several light system covering different regions of the UV and visible spectrum was equipped in the chamber to simulate the atmospheric photochemistry, including a Krypton-Fluoride excimer UV-laser (3 W, λ = 248 nm), two UV LEDs (2 × 16.5 W, λ = 370–390 nm) and four Hamamatsu Xenon arc lamps (4 × 200 W, λ = 250–580 nm)^9,43^. For varying NO/NO_2_ experiment run during CLOUD11, an additional UV-sabre (400 W UVS3, centered on 385 nm) system was installed to further photolyze NO_2_ into NO. The NO/NO_2_ ratio can be adjusted by changing the UV-sabre light intensity. The NO_2_ photolysis frequency, jNO_2_, was measured by NO_2_ actinometry and varying the UV-sabre intensity.

### Experimental design

Our objective of this study is to investigate the role of NO_x_ in the oxidation of monoterpene and HOM formation in the atmospherically relevant concentrations (low NO_x_ environment). The chamber conditions were kept stable at the temperature of 278 K and relative humidity of 38%. Ozone was injecting at a constant rate to keep the concentration around 40 ppbv. The monoterpene used in this study is a mix of α-pinene and Δ−3-carene with a volume mixing ratio of 2:1, which represents the condition at our reference site of SMEAR II station in southern Finland^29,30^. The steady-state concentration of monoterpene mixture was set to 300 pptv, 600 pptv and 1200 pptv. In this study, with the target to understand the role of NO_x_ in detail, we conducted three different experiment runs, including pure NO_2_ run, constant NO/NO_2_ ratio runs and, varying NO/NO_2_ run. Pure NO_2_ run and constant NO/NO_2_ ratio runs were conducted during November of 2015 (CLOUD10 campaign). The varying NO/NO_2_ experiment was performed in November of 2016 (CLOUD11 campaign). NO_2_ was injected into the chamber under dark condition during pure NO_2_ run to maintain a NO free environment. NO was injected into the chamber for constant NO/NO_2_ runs, and mostly oxidized to NO_2_ by reacting with O_3_. For each experiment run, monoterpene was injected into the chamber to get its steady-state concentration before injecting NO_x_. The steady-state concentrations of NO_x_ were set to 0, 0.7, 1.9 and 4.6 ppbv for the pure NO_2_ run and constant NO/NO_2_ ratio runs, representing very clean to slightly polluted environments, and at approximately 1 ppbv for the varying NO/NO_2_ experiment to represent the mean value at the SMEAR II station. Throughout all the NO/NO_2_ experimental runs, the UV LEDs light system was kept on to photolyze some NO_2_ to NO. The ratio of NO/NO_2_ was kept at ~0.7% in constant NO/NO_2_ experimental runs. Noting that due to the titration of NO in the NO injection run (constant NO/NO_2_ experiment), the steady state concentration of O_3_ decreased slightly from 40 ppbv at 0 ppbv NO_x_ to around 36 ppbv at 4.6 ppbv NO_x_. During the varying NO/NO_2_ experimental run, NO concentration was adjusted from 0 pptv to 82 pptv by changing the intensity of UV-sabre.

### Measurements

Measurement of gas-phase HOM and OH radicals. Concentrations of highly oxygenated molecules (HOM) were measured with a nitrate-ion based chemical ionization atmospheric pressure interface time-of-flight mass spectrometer (CI-APi-TOF)^44^. The nitrate ions are produced by exposing nitric acid (HNO_3_)-containing sheath flow to soft x-ray radiation. The sulfuric acid and targeted HOM are charged in the drift tube before entering to the APi and analyzing in the TOF chamber. To quantify HOM, the system was calibrated and corrected using the methods similar to our previous work^9,10^. Since the loss rate of sulfuric acid is well characterized, the concentration and calibration coefficient can be obtained. This calibration coefficient was also used to quantify HOM with the consideration of weight-dependent ion transmission^45^ and sampling loss in the inlet. We processed the raw data using the MATLAB tofTools package (version 603)^44^. High-resolution analysis was used to identify the peaks with different elemental formulae.

OH concentrations in the chamber were determined by the following equation.

1 $$[{{{{{\rm{OH}}}}}}]=\frac{{{{{{{\rm{k}}}}}}}_{{{{{{\rm{loss}}}}}}}[{{{{{\rm{SA}}}}}}]}{{{{{{{\rm{k}}}}}}}_{{{{{{\rm{SO}}}}}}2+{{{{{\rm{OH}}}}}}}\left[{{{{{\rm{OH}}}}}}\right]\times [{{SO}}_{2}]}$$

Where k_loss_ refers to the loss of sulfuric acid to the aerosol particles (condensation sink) and to the wall (wall loss); k_SO2+OH_ refers to the reaction rate constant between OH and SO_2_ at 278 K. OH concentrations were also simulated by the ADCHAM model. The simulated concentration was identical to the estimated concentration via Eq. (1). NO_3_ concentrations were calculated by the change in monoterpene concentration after adjusting NO_x_ concentration at each experimental steps.

Measurement of monoterpenes and trace gases. A newly developed proton transfer reaction time-of-flight mass spectrometer, named PTR3, was deployed to measure the concentrations of monoterpenes and other VOCs^46^. Since α-pinene and Δ−3-carene fragment differently in the instrument, they can be calibrated individually. Sulfur dioxides (SO_2_) and ozone (O_3_) were measured using Thermo Scientific gas monitors (model 42i for SO_2_ and model 49i for O_3_). In view of the low concentration of nitrogen oxide (NO), an advanced NO monitor (ECO PHYSICS, model: CLD 780 TR) was used to measure NO accurately. The detection limit of this NO monitor is 3 pptv for 1-min integration time. Nitrogen dioxide (NO_2_) was measured using a cavity-attenuated phase-shift nitrogen dioxide monitor (CAPS NO_2_, Aerodyne Research Inc.) close to the injection of the chamber and by Cavity-Enhanced Differential Optical absorption spectroscopy (CE-DOAS) at the top of the chamber. NO and NO_2_ were injected at the bottom of the chamber close to a mixing fan and quickly dispersed into the chamber. NO_2_ measurements at the top and bottom of the chamber suggest the NO_2_ concentrations to be well mixed, with concentrations at the top and bottom typically not differing by more than 25%. All the gases were sampled from the middle of the chamber. For the NO_2_ injection runs, including the pure NO_2_ experiment run and the varying NO/NO_2_ experiment run, all the trace gases were homogeneous distributed in the chamber. For the constant NO/NO_2_ experiment runs, the NO mixing ratio close to its injection port was about 5 times higher than that in the middle of the chamber. However, due to the fast reaction of NO and O_3_, the space with highly concentrated NO should be much smaller than the overall chamber volume.

Ambient measurement campaigns of HOM. Ambient HOM measurement data from two field campaigns were used to verify the role NO_x_ in HOM formation in the real atmosphere. One campaign was conducted at a boreal forest site (SMEAR II station) in southern Finland between 15 and 24 May 2013 to represent the low-NO_x_ environment^11^, and the other was at an urban site in east China (SORPES station) during 2 August and 6 September 2019 to represent the high-NO_x_ environment^22^. Detailed description of the campaigns can be found in Roldin et al., 2019^11^ and Liu et al., 2021^22^. In short, a CI-APi-HTOF at SMEAR II and a CI-APi-LTOF at SORPES were used to measure HOM and sulfuric acid. VOCs precursors were measured by a proton transfer reaction mass spectrometer (PTR-MS, Ionicon). NO_x_ and other trance gases (e.g., O_3_) were measured by TEI gas analyzers.

### Positive matrix factorization (PMF)

PMF is a widely used receptor model for source apportionment analysis^47^. In this study, we first preformed the high-resolution peak fitting for the varying NO/NO_2_ experiment run, during which an LToF based nitrate CIMS was deployed with the resolution up to ~10,000 Th/Th that allowed us to identify the peaks more accurate. Then, we prepared the data matrix and error matrix according to the methods described by Yan et al.^17^ for the PMF model input. An IGOR based analyzing interface SoFi (solution finder, version6.3) and ME-2 was used to perform the high-resolution PMF analysis^48^. A detailed description of PMF used for a nitrate-CIMS based dataset can be found in our previous study^17^. 5 factors resolved from the PMF analysis provide the optimal solutions. We shortly introduce the 5 factors as follows.

Δ−3-carene ozonolysis factor (D3C factor): 400 pptv Δ−3-carene was injected into the chamber as the first run-step, during which only carene and ozone reacted in the chamber. Therefore, PMF resolved the Δ−3-carene ozonolysis factor as the first factor, the spectrum of which is a bit different from the well-known α-pinene ozonolysis factor (Supplementary Fig. 3)^6^. The pre-dominant molecule is C_10_H_14_O_9_, followed by two RO_2_ radicals of C_10_H_15_O_8_ and C_10_H_15_O_10_. Dimer formation at this stage is not significant, due to the lower concentration of precursors which that limit the RO_2_ cross-reactions.

α-pinene and Δ−3-carene ozonolysis factor (AP + D3C factor): 800 pptv α-pinene was added after the Δ−3-carene step reaching the steady state to obtain the targeted mixture of monoterpenes. PMF identified this system as a pure ozonolysis factor, the spectrum of which is very similar to Nighttime Factor 1 resolved from field observation at SMEAR II station (Fig. S4)^17^. Compared to the D3C factor, the intensity of C_10_H_14_O_7_ increased dramatically and was to be one of the main fingerprint molecules of the AP + D3C factor. In contrast, RO_2_ radicals (e.g., C_10_H_15_O_8_ and C_10_H_15_O_10_) decreased significantly, probably due to the enhanced RO_2_ cross-reactions which consumed RO_2_ radicals but promoted the dimer formations, e.g., C_19_H_28_O_11_ and C_20_H_32_O_11_. In addition, the intensity of OH-initiated HOM molecules, e.g., C_10_H_16_O_7_ and C_10_H_16_O_9_, enhanced significantly. It could be that OH production from monoterpene ozonolysis was elevated, or the HOM yield from OH oxidation of α-pinene was higher than OH oxidation of Δ−3-carene.

NO_3_ oxidation factor (NO_3_ factor): Around 1 ppb NO_2_ was injected into the chamber after the above two steps. NO_2_ reacted with O_3_ to form NO_3_ radical, which oxidized monoterpenes to be an additional HOM source. Therefore, PMF resolved an NO_3_ factor aside from the pure ozonolysis factor at this stage. The spectrum of the NO_3_ factor was similar to that of the Nighttime Factor 2 resolved from field observation at SMEAR II station (Fig. S4)^17^. CHON monomers of C_10_H_15_O_8,10_N and dimers of C_20_H_31_O_11,13_N were the major fingerprint molecules. A significant formation of C_10_H_16_O_10_N_2_ was observed at this stage (in the NO_3_ factor), during which only NO_3_ and NO_2_ presented as the N-containing precursor gases. This is different from previous understanding that CHON_2_ can be only formed from a CHON radical produced from NO_3_ oxidation and terminated by NO. One possible explanation is that C_10_H_16_O_10_N_2_ in this factor is acyl nitrate formed via the reaction of an acylperoxy radical with NO_2_.

NO involved NO_3_ oxidation factor (NO_3_ + NO factor): After producing NO by photolyzing NO_2_ in the chamber, a new factor was resolved and defined as NO_3_ + NO factor (Supplementary Fig. 3). This factor is a transition factor from the NO_3_ factor to a NO heavily participating factor (NO factor). A bunch of CHON molecules, e.g., C_10_H_15_O_7_N and C_10_H_15_O_9_N, were formed from the reaction between RO_2_ radical and NO. Additional CHON_2_ molecules other than C_10_H_16_O_10_N_2_, e.g., C_10_H_16_O_9_N_2_, were formed from the NO_3_-initiated CHON radical reacting with NO. Dimer formation became very week. Fragmented molecules (e.g., C_5_H_6_O_7_), as well as C_15_ HOM molecules (e.g., C_15_H_17_O_9,10_), started to appear in this factor because of the NO, which can react with RO_2_ to form RO to enhance the possibility of fragmentation.

NO heavily participating factor (NO factor): When further stepping up the NO concentration, PMF resolved another factor, in which NO participated the HOM formation heavily (Supplementary Fig. 3). In this factor, a considerable amount of fragmented molecules (e.g., C_5_H_6_O_5_, C_5_H_6_O_7_, C_7_H_11_O_8_, and C_8_H_13_O_9_N) were observed. HOM dimers almost disappeared, suggesting the reaction with NO was becoming the dominating fate of RO_2_ instead of its cross-reaction.

In summary, optimal PMF solutions provide 4 types of factors for all the four experimental runs, including two ozonolysis factors (D3C factor and MT factor), an NO_3_ oxidation factor (NO_3_ factor), an NO involved NO_3_ oxidation factor (NO_3_ + NO factor) and a NO heavily-participating factor (NO factor). Factors appear successively from the D3C factor to the NO factor accompanying with stepping up of NO_2_ or NO. The spectrum of HOM changes as follows: it is dominated by typical CHO HOM in the ozonolysis factors, but switches to CHON monomers (e.g., C_10_H_15_O_8,10_N) and dimers (e.g., C_20_H_31_O_11,13_N) in NO_3_ factor. In the NO_3_ + NO factor, CHON (e.g., C_10_H_15_O_7-10_N) dominates and CHON_2_ (e.g., C_10_H_16_O_9,10_N_2_) appears extensively. Finally, a significant amount of fragmented molecules (e.g., C_5_H_6_O_5_, C_5_H_6_O_7_, C_7_H_11_O_8_) appears in the NO factor. HOM dimers decreases gradually and almost disappear in the NO factor. This is in accordance with our previous understanding that CHON radicals and CHON dimers can be only formed from the NO_3_ involved reaction chain; most CHON_2_ are only formed from a CHON radical produced from NO_3_ oxidation and terminated by NO, except for some acyl nitrate formed via the reaction of an acylperoxy radical with NO_2_ (e.g., C_10_H_16_O_10_N_2_).

### ADCHEM model

We use the Aerosol Dynamics gas- and particle-phase chemistry model for laboratory CHAMber studies (ADCHAM)^49^ and the trajectory model for Aerosol Dynamics gas- and particle-phase CHEMistry and radiative transfer (ADCHEM)^50^. They share a detailed gas-phase kinetic code that combines peroxy radical autoxidation mechanism (PRAM) and Master Chemical Mechanism version 3.3.1 (MCMv3.3.1) using the Kinetic PreProcessor (KPP)^51^. As described in Roldin et al., 2019^11^, PRAM explicitly simulates the formation of peroxy radicals (RO_2_) and their oxidation products. We provide the yield, branch ratio, and autoxidation rate constants of main chemical reactions in Supplementary Table 3. Given that the mechanism of fragmentation chemistry is not well understood, ADCHAM tends to underestimate the fragments, the production of which is accompanied by an increase in the NO/MT ratio. Therefore, the model slightly underestimates the HOM yield in the highest NO_x_ step of the constant NO/NO_2_ experiment with 300 pptv monoterpene (Fig. 1c).

In this work, we added the following mechanisms into the model to improve the simulation.

1. NO_3_ chemistry in HOM formation, which included RO_2_ formed when monoterpenes are oxidized by NO_3_ and RO formed via RO_2_-NO_3_ reactions.
2. Terminal reaction by bimolecular reactions between NO_2_ and certain RO_2_ (e.g., C_10_H_15_O_5_)
3. Various isomers of RO_2_ were considered, which greatly reduced the uncertainty of simulating responses that are highly dependent on RO_2_ structures.
4. Refine the representation of RO isomerization

Since HOM have very low saturation vapor pressures, we assume that they are lost losses irreversibly onto the chamber wall, and calculate the wall loss from the following equation^52^:

2 $${{{{{{\rm{k}}}}}}}_{{{{{{\rm{wall}}}}}}}=\,{{{{{{\rm{C}}}}}}}_{{{{{{\rm{wall}}}}}}}\sqrt{{{{{{\rm{D}}}}}}}$$

where C_wall_ is an empirical parameter, which is 0.0075 cm^−1^ s^−0.5^ derived from dedicated sulfuric acid decay experiments at 5 °C. The diffusion coefficients D_i_ for each HOM_i_ are approximated with the equation ${{{{{{\rm{D}}}}}}}_{{{{{{\rm{i}}}}}}}({{{{{{\rm{cm}}}}}}}^{2}{{{{{{\rm{s}}}}}}}^{-1})=0.31\bullet {{{{{{\rm{M}}}}}}}_{{{{{{\rm{i}}}}}}}^{-1/3}$, where M_i_ is the mass of molecule with the unit of g mol^−1^.

### HOM yield calculation

HOM yield calculation in CLOUD chamber and SMEARII station: we defined the yield of HOM as the fraction of reacted monoterpenes that produced HOM. Due to the complex formation of HOM through multiple oxidations, the production rate of HOM was replaced by the HOM loss rate under steady state:

3 $${{{{{\rm{MT}}}}}}-{{{{{\rm{HOM\; yield}}}}}}=\frac{{{{{{{\rm{k}}}}}}}_{{{{{{\rm{loss}}}}}}}[{{{{{\rm{HOM}}}}}}]}{{{{{{{\rm{k}}}}}}}_{{{{{{\rm{MT}}}}}}+{{{{{\rm{oxidants}}}}}}}\left[{{{{{\rm{oxidiants}}}}}}\right]\times [{{{{{\rm{MT}}}}}}]}$$

where oxidants consist of O_3_, OH, and NO_3_ in this study and k_loss_ is the total loss rate of HOM to the chamber walls in CLOUD experiments, to the ground or other surfaces (Dry deposition) and aerosol particles (condensation) in field observations. We propagate the error for each chamber experiment with four different NO_x_ levels. Within each selected time windows, absolute errors of [HOM], [SO_2_], [sulfuric acid], CS, [O_3_], [α-pinene], [Δ−3-carene] are derived from the measurements as the 3-sigma standard deviation. Since NO_3_ concentrations are calculated based on the consumed monoterpene concentration, they share the same relative error with [α-pinene] and [Δ−3-carene]. For the loss of HOM, we assume zero error for k_wall_ and k_dilu_. The propagated error of the HOM yield varies slightly within 6–8% among different experiments (Fig. 1).

When calculating condensation for HOM measured at SMEAR II, pure liquid saturation vapor pressures(*p*_*0*_) were calculated based on saturation concentrations (C_sat_), which can be parameterized by the numbers of carbon(n_C_), oxygen(n_O_), and nitrogen(n_N_) atoms^53^:

4 $${{{\log }}}_{10}\left({{{{{{\rm{C}}}}}}}_{{{{{{\rm{sat}}}}}}}\right)=\left({{{{{{\rm{n}}}}}}}_{0}-{{{{{{\rm{n}}}}}}}_{{{{{{\rm{C}}}}}}}\right){{{{{{\rm{b}}}}}}}_{{{{{{\rm{C}}}}}}}-\left({{{{{{\rm{n}}}}}}}_{{{{{{\rm{O}}}}}}}-3{{{{{{\rm{n}}}}}}}_{{{{{{\rm{N}}}}}}}\right){{{{{{\rm{b}}}}}}}_{{{{{{\rm{O}}}}}}}-2\frac{\left({{{{{{\rm{n}}}}}}}_{{{{{{\rm{O}}}}}}}-3{{{{{{\rm{n}}}}}}}_{{{{{{\rm{N}}}}}}}\right){{{{{{\rm{n}}}}}}}_{{{{{{\rm{C}}}}}}}}{\left({{{{{{\rm{n}}}}}}}_{{{{{{\rm{C}}}}}}}+{{{{{{\rm{n}}}}}}}_{{{{{{\rm{O}}}}}}}-3{{{{{{\rm{n}}}}}}}_{{{{{{\rm{N}}}}}}}\right)}{{{{{{\rm{b}}}}}}}_{{{{{{\rm{CO}}}}}}}-{{{{{{\rm{n}}}}}}}_{{{{{{\rm{N}}}}}}}{{{{{{\rm{b}}}}}}}_{{{{{{\rm{N}}}}}}}$$

where n_0_ = 25, b_C_ = 0.475, b_O_ = 0.2, b_CO_ = 0.9, and b_N_ = 2.5, respectively.

As volatilities of organic molecules detected span over a wide range, we grouped the hundreds of organic molecules detected in the chamber into different bins within a volatility basis set (VBS)^54^. Then we can calculate the HOM mass yield via formula (3), in which monoterpene concentrations were measured, HOM was also measured and grouped into a series of bins, k_loss_, OH and NO_3_ radical were simulated by the model. To minimize the potential uncertainty, we selected HOM species with C*(300 K) below 3×10^−4^ μg m^−3^ as those would condense onto aerosol particles irreversibly.

HOM yield calculation at SORPES station: In polluted east China, where VOCs species distribution and photochemistry are far more complex than in the boreal environment, it is challenging to simulate HOM concentrations. Especially during nighttime, when the NO concentration is very high and consume most known oxidants, e.g., NO_3_ radical and O_3_. Therefore, we only calculated the daytime HOM yield through the following Eq. (5):

5 $${{{{{\rm{MT}}}}}}-{{{{{\rm{HOM\; yield}}}}}}=\frac{{[{{{{{\rm{ELVOCs}}}}}}]}_{{{{{{\rm{MT}}}}}}}\cdot {{{{{\rm{CS}}}}}}}{{{{{{{\rm{k}}}}}}}_{{{{{{\rm{MT}}}}}}+{{{{{\rm{OH}}}}}}}\left[{{{{{\rm{MT}}}}}}\right]\cdot \left[{{{{{\rm{OH}}}}}}\right]+{{{{{{\rm{k}}}}}}}_{{{{{{\rm{MT}}}}}}+{{{{{{\rm{O}}}}}}}_{3}}\left[{{{{{\rm{MT}}}}}}\right]\cdot \left[{{{{{{\rm{O}}}}}}}_{3}\right]}$$

Here, ${[{{{{{\rm{ELVOCs}}}}}}]}_{{{{{{\rm{MT}}}}}}}$ is the concentration of monoterpene-derived ELVOCs, $\left[{{{{{\rm{MT}}}}}}\right]$ is the concentration of monoterpenes. Monoterpene derived HOM are selected as HOM molecules with a carbon number of 10 and a double bond equivalent (DBE) number between 2 and 4^8^. Since the atmospheric oxidation capacity in polluted east China is usually strong, HOM are possibly produced through multi-generational oxidation. We estimated the contribution of multi-generation by the following method, and excluded it from the yield calculation. First, dinitrates (HOM molecules with two nitrate groups) are regarded as the multi-generational products, since one oxidation step can only add one nitrate group to the product molecule; Second, HOM molecules with a DBE of 2 are produced either from OH-initiated oxidation of monoterpenes or from the multi-generational oxidation process of monoterpenes. The calculated contribution of multi-generational oxidation to monoterpene HOM ranges from 15.8% to 33.5%. Here, we only selected non-nitrates and mononitrates with a DBE of 3−4 to calculate the yield to exclude the effects of multi-generational oxidation (Supplementary Fig. 7).

Molecules with C*(300 K) of below 3×10^−4^ μg m^−3^ were further selected as ELVOCs those can condense onto aerosol particles irreversibly. Since the oxidation products with higher volatility are not included due to their long lifetimes cause the steady-state assumption not to hold, the HOM yield calculated here, which is considerably under-estimated, can be considered as the low limit. The OH concentration was calculated by applying the Eq. (1), based on the assumption that gaseous sulfuric acid is mostly produced from the oxidation of SO_2_ by OH and primarily lost by condensing onto particles. ${{{{{{\rm{k}}}}}}}_{{{{{{\rm{OH}}}}}}+{{{{{{\rm{SO}}}}}}}_{2}}$ is a termolecular reaction constant for the rate-limiting step of the formation pathway of H_2_SO_4_ at the temperature of the sampling time in the atmosphere^55^, and k_loss_ is the loss rate of H_2_SO_4_ by condensation to aerosol surface (CS, condensation sink). Although reactions of SO_2_ with products from the ozonolysis of alkenes generate a moderate amount of nighttime sulfuric acid, with little effect on daytime sulfuric acid^56^. This is also one reason that we calculated the yield of MT-HOM only during the daytime in this study. The error of OH does not change the relative distribution of RO_2_ from different precursors.

In the atmosphere without direct anthropogenic emissions, NO concentrations, mainly from the photolysis of NO_2_, show a clear diurnal cycle from almost zero during the night to its maximum before noontime. For example, NO concentration and NO to NO_2_ ratio revealed its peak value at around 10:00–11:00 am at our reference station of SMEAR II^17^. Correspondingly, the HOM yield will vary non-linearly and diurnally. Therefore, the HOM yield is determined by the ratio of NO/NO_2_ at a specific time of the day. We thus take the daytime average HOM yield as the HOM yield at fixed NO_x_ and monoterpene concentration. We conducted a series of simulations with monoterpene concentration varying from 100 to 1800 pptv, and NO_x_ concentration from 0.01 to 100 ppb. With the assumption NO is solely formed from NO_2_ photolysis, the NO/NO_2_ ratio varied to follow the observed diurnal cycle at SMEAR II station from almost zero at night to 14.3% at 10 am. The average daily dependence of HOM on monoterpene and NO_x_ concentrations can be estimated on the basis of integration with this function. We then take the daily average HOM yield as the HOM yield at fixed NO_x_ and monoterpene concentration, and illustrated in Fig. 4.

## Supplementary information

Supplementary Information

## Data Availability

The observation data that support the main findings of this study are available at figshare (10.6084/m9.figshare.22724648.v1)^57^.

## Code availability

Data processing techniques are available on request from the corresponding author.
