# Supplementary material for: NO at low concentration can enhance the formation of highly oxygenated biogenic molecules in the atmosphere
Source: Nat Commun. 2023 Jun 8;14:3347. doi: 10.1038/s41467-023-39066-4 (PMC10250349; doi:10.1038/s41467-023-39066-4)
Supplement: Supplementary file 1 — Supplementary Information [file 41467_2023_39066_MOESM1_ESM.pdf]

Supplementary Information for

NO at low concentration can enhance the formation of highly oxygenated biogenic molecules in the atmosphere

Wei Nie[1,2,3† *], Chao Yan[1,2,3†], Liwen Yang[1], Pontus Roldin[4, 5], Yuliang Liu[1], Alexander L. Vogel[6], Ugo Molteni[7,8,9], Dominik Stolzenburg[3,10], Henning Finkenzeller[11], Antonio Amorim[12], Federico Bianchi[3], Joachim Curtius[6], Lubna Dada[3,7], Danielle C. Draper[8,30], Jonathan Duplissy[3,13], Armin Hansel[14], Xu-Cheng He[3], Victoria Hofbauer[15], Tuija Jokinen[3,16], Changhyuk Kim[17, 18], Katrianne Lehtipalo[3,19], Leonid Nichman[20], Roy L. Mauldin[15,21], Vladimir Makhmutov[22,23], Bernhard Mentler[24], Andrea Mizelli-Ojdanic[10,31], Tuukka Petäjä[3], Lauriane L. J. Quéléver[3], Simon Schallhart[3,19], Mario Simon[6], Christian Tauber[10], António Tomé[25], Rainer Volkamer[11], Andrea C. Wagner[6, 32], Robert Wagner[3], Mingyi Wang[18], Penglin Ye[26], Haiyan Li[27], Wei Huang[3], Ximeng Qi[1,2], Sijia Lou[1], Tengyu Liu[1,2], Xuguang Chi[1, 2], Josef Dommen[7], Urs Baltensperger[7], Imad El Haddad[7], Jasper Kirkby[28], Douglas Worsnop[3,29], Markku Kulmala[1,3], Neil M. Donahue[15], Mikael Ehn[3], Aijun Ding[1, 2*]

Affiliations:

[1] Joint International Research Laboratory of Atmospheric and Earth System Research, School of Atmospheric Sciences, Nanjing University, Nanjing, China

[2] National Observation and Research Station for Atmospheric Processes and Environmental Change in Yangtze River Delta, Nanjing, Jiangsu Province, China

[3] Institute for Atmospheric and Earth System Research / Physics, Faculty of Science, University of Helsinki, Helsinki, Finland

[4] Department of Physics, Lund University, P. O. Box 118, SE-221 00 Lund, Sweden

[5] IVL, Swedish Environmental Research Institute, SE-211 19, Malmö, Sweden

[6] Institute for Atmospheric and Environmental Sciences, Goethe University Frankfurt, Frankfurt am Main, 60438, Germany

[7] Laboratory of Atmospheric Chemistry, Paul Scherrer Institute, 5232, Villigen PSI, Switzerland

[8] Department of Chemistry, University of California, Irvine, CA, 92697, USA

[9] Forest Dynamics, Swiss Federal Institute for Forest, Snow and Landscape Research, 8903 Birmensdorf, Switzerland

[10] Faculty of Physics, University of Vienna, Boltzmanngasse 5, 1090, Vienna, Austria

[11] Department of Chemistry & CIRES, University of Colorado Boulder, Boulder, CO, 80309, USA

[12] CENTRA and FCUL, Universidade de Lisboa, Campo Grande, 1749-016, Lisboa, Portugal

[13] Helsinki Institute of Physics (HIP)/Physics, Faculty of Science, University of Helsinki, 00014, Helsinki, Finland

[14] Institute of Ion and Applied Physics, University of Innsbruck, 6020, Innsbruck, Austria
[15] Center for Atmospheric Particle Studies, Carnegie Mellon University, Pittsburgh, PA, USA
[16] Climate & Atmosphere Research Centre (CARE-C), The Cyprus Institute, P.O. Box 27456, Nicosia, CY-1645, Cyprus
[17] School of Civil and Environmental Engineering, Pusan National University, Busan, 46241, Republic of Korea
[18] Division of Chemistry and Chemical Engineering, California Institute of Technology, Pasadena, CA, 91125, USA
[19] Finnish Meteorological Institute, Erik Palménin aukio 1, 00560, Helsinki, Finland
[20] Flight Research Laboratory, National Research Council Canada, Ottawa, K1A 0R6, ON, Canada
[21] Department of Atmospheric and Oceanic Sciences, University of Colorado Boulder, Boulder, CO, USA
[22] P.N. Lebedev Physical Institute of the Russian Academy of Sciences, 53, Leninskiy Prospekt, Moscow, Russian Federation
[23] Moscow Institute of Physics and Technology (National Research University), 1A Kerchenskaya st., Moscow, Russian Federation
[24] Ion Molecule Reactions & Environmental Physics Group Institute of Ion Physics and Applied Physics Leopold-Franzens University, Innsbruck Technikerstraße 25, A-6020, Innsbruck, Austria
[25] IDL-Universidade da Beira Interior, Rua Marquês D'Ávila e Bolama, 6201-001, Covilhã, Portugal
[26] Shanghai Key Laboratory of Atmospheric Particle Pollution and Prevention (LAP3), Department of Environmental Science and Engineering, Fudan University, Shanghai, 200438, China
[27] School of Civil and Environmental Engineering, Harbin Institute of Technology, Shenzhen, 518055, China
[28] CERN, CH-1211, Geneva, Switzerland
[29] Aerodyne Research Inc., Billerica, MA, 01821, USA
[30] Now at: Division of Geological and Planetary Sciences, California Institute of Technology, Pasadena, CA, 91125, USA
[31] Now at: Department of Chemistry & CIRES, University of Colorado Boulder, Boulder, CO, 80309, USA
[32] Now at: Faculty of Industrial Engineering, FH Technikum Wien - University of Applied Sciences, 1200 Vienna, Austria
† These authors contributed equally
*Correspondence to: Wei Nie (niewei@nju.edu.cn) and Aijun Ding (dingaj@nju.edu.cn)

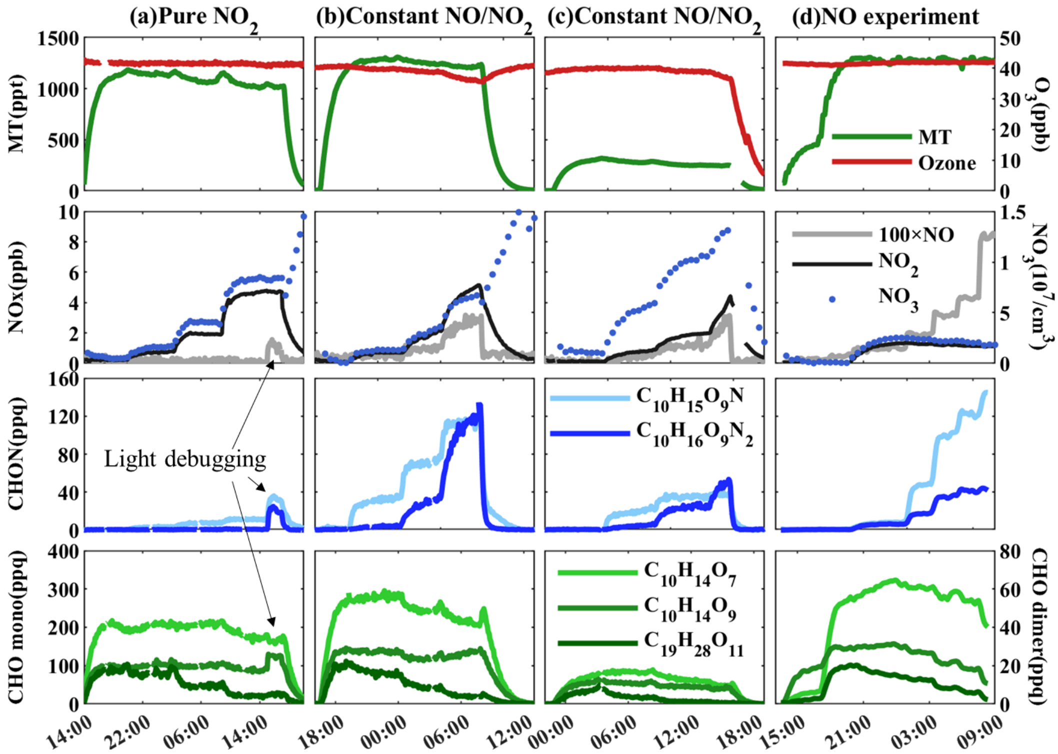

Supplementary Figure 1 Example CLOUD experiments for (a) pure NO_2 with 1200 ppt monoterpene, (b) constant NO/NO_2 ratio with 1200 ppt monoterpene, (c) constant NO/NO_2 ratio with 300 ppt monoterpene and (d) varying NO/NO_2 ratio with 1200 ppt monoterpene. In the first 3 experiments, α-pinene and Δ-3-carene were mixed in a 2:1 ratio before injecting into the chamber. In the last run, Δ-3-carene was injected into the chamber for about 1 hour before adding α-pinene. The top row of panels shows monoterpene and ozone concentrations during the four experiments; NO, NO_2 and NO_3 are shown in the second row; two example CHON molecules ($C_{10}H_{15}O_9N$ and $C_{10}H_{16}O_9N_2$) are shown in the third row; and two CHO monomer molecules ($C_{10}H_{14}O_7$ and $C_{10}H_{14}O_9$) and one CHO dimer molecule ($C_{20}H_{32}O_{11}$) are shown in the bottom row. The small peak in the pure NO_2 experiment near 14:00 is due to debugging of the light system, during which a small amount of NO_2 was photolyzed to NO.

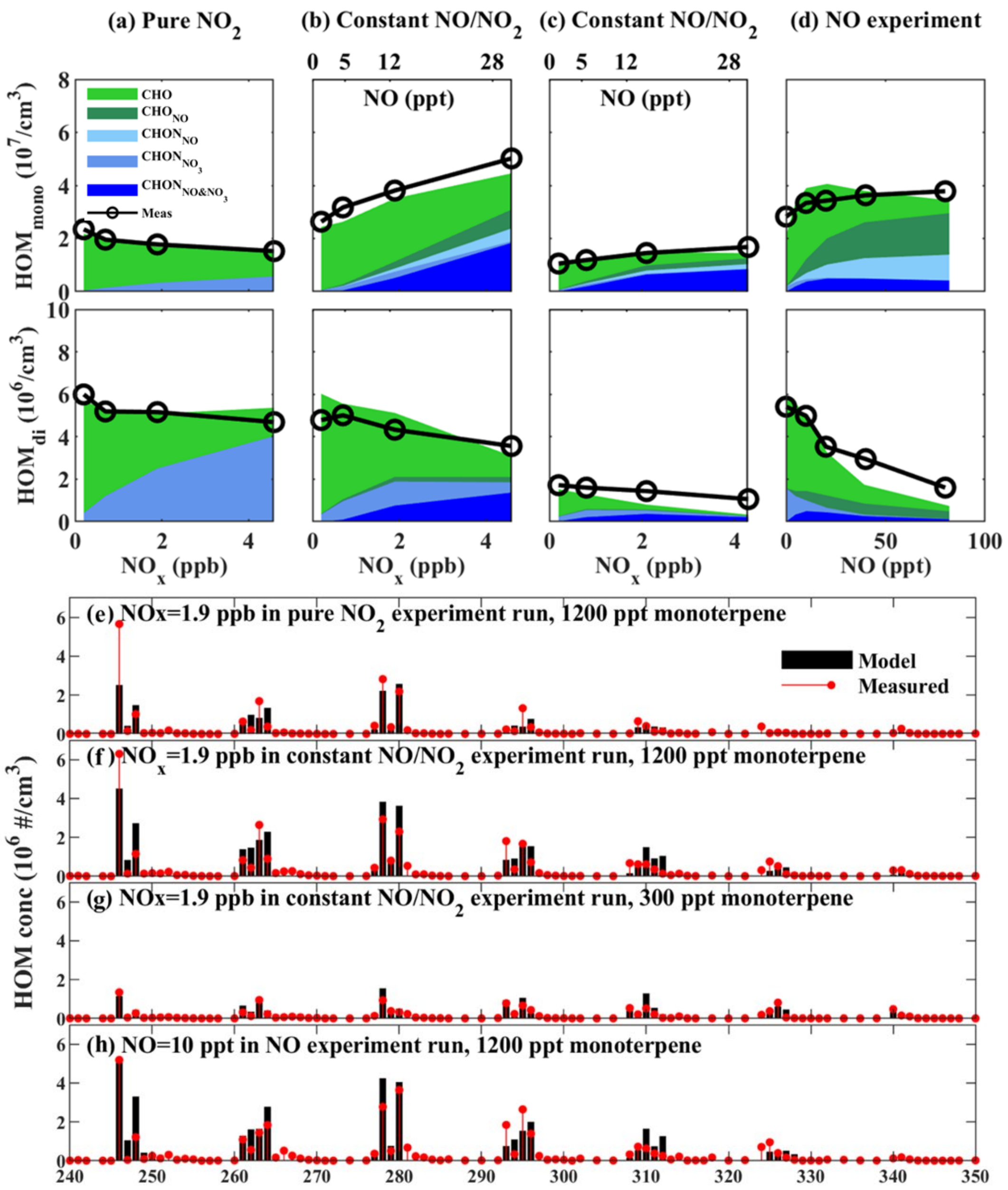

Supplementary Figure 2 Detailed comparisons on the concentration (a-d) and spectrums (e-h) between modeled and observed highly oxygenated organic molecules (HOMs) in (a and e) pure NO_2 experiment run with 1200 ppt monoterpene, (b and f) constant NO/NO_2 ratio experiment run with 1200 ppt monoterpene, (c and g) constant NO/NO_2 ratio experiment run with 300 ppt monoterpene and (d and h) varying NO/NO_2 ratio experiment run with 1200 ppt monoterpene. CHO and CHO_{NO} molecules are denoted in light and dark green; $CHON_{NO}$, $CHON_{NO3}$ and $CHON_{NO\&NO3}$ molecules are denoted in colors from light blue to dark blue. The measured data points (a-d) and spectrums (e-h) are half-hour averaged value of each experimental step reaching steady-state equilibrium.

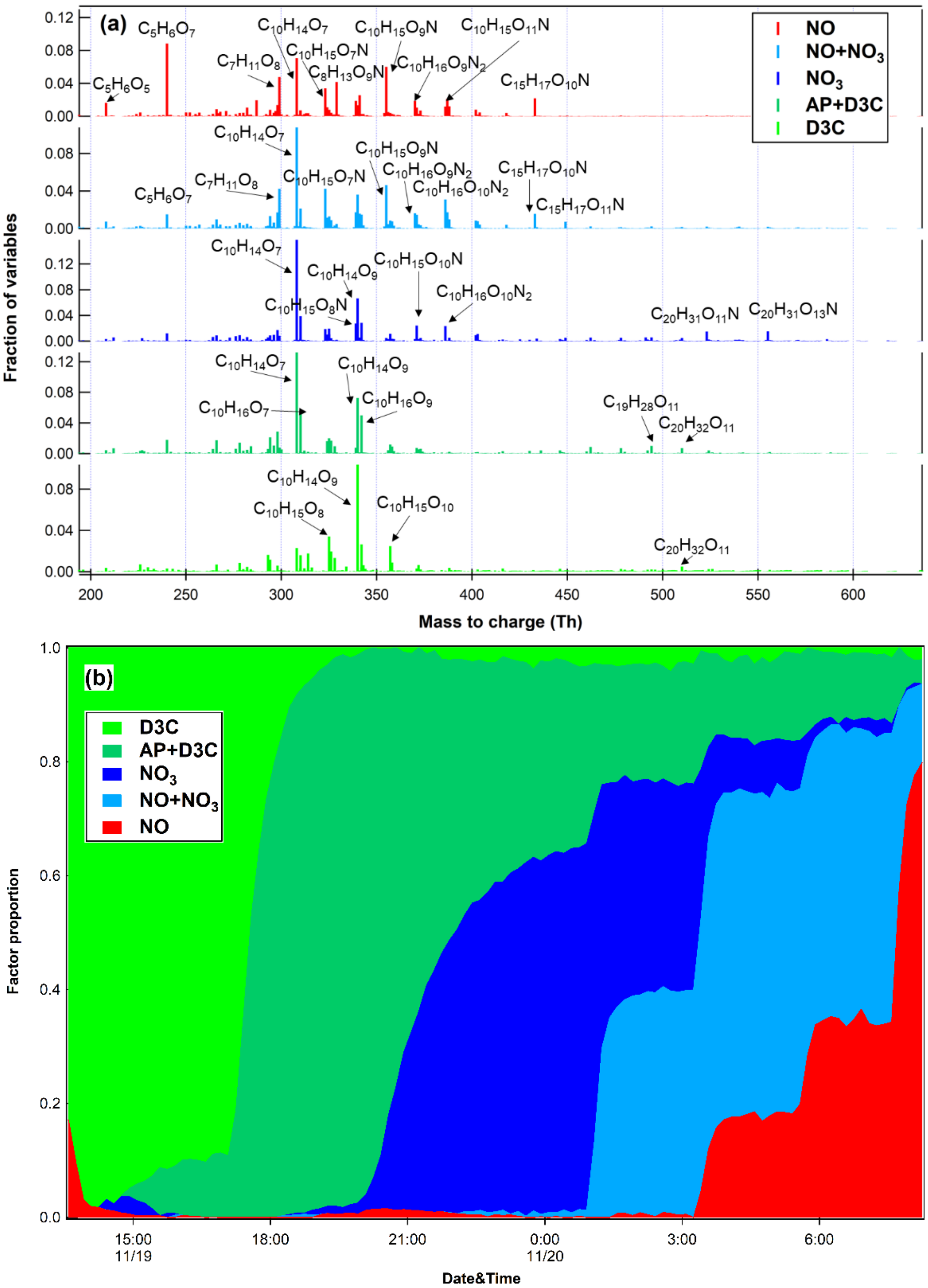

Supplementary Figure 3 High Resolution PMF results of the varying NO/NO2 ratio experiment run with 1200 ppt monoterpene. (a) Spectrums of the five PMF factors. The total signal of each factor is normalized to unity, and y axis is the fraction of variables in the factor in percentage; (b) Proportion of factors at different experiment steps.

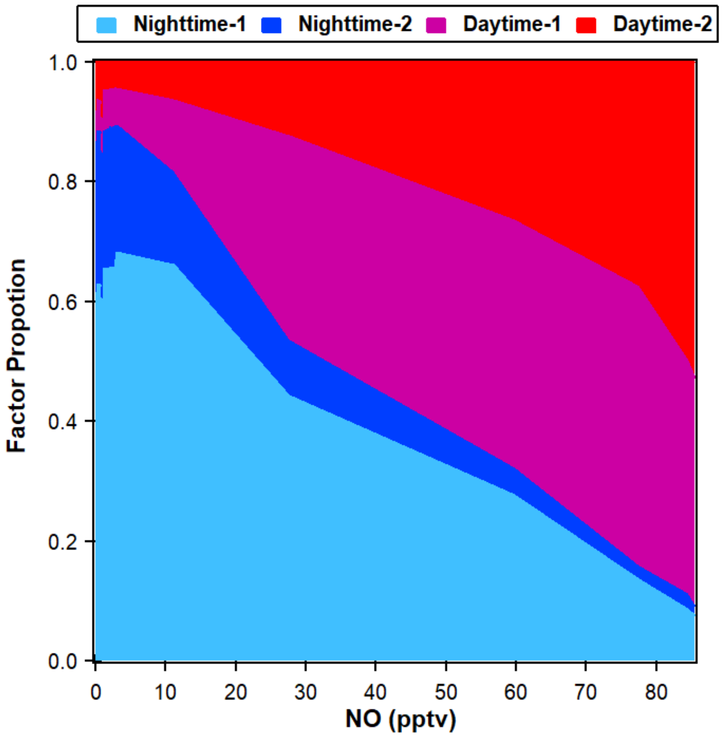

Supplementary Figure 4 Dependences of 4 monoterpene related factors on NO concertation during the observation at SMEAR II station in southern Finland from 4 April to 8 May 2012[17]. Nighttime-1 factor refers to the ozonolysis factor; Nighttime-2 factor refers to the NO_3 factor; Daytime-1 factor primarily refers to NO_3 + NO factor; Daytime-2 factor primarily refers to NO factor. NO_x involved factors contributed around 35% of the monoterpene imitated HOMs in NO-free environment during nighttime, and increased to more than 90% with 80-90 ppt NO at noontime.

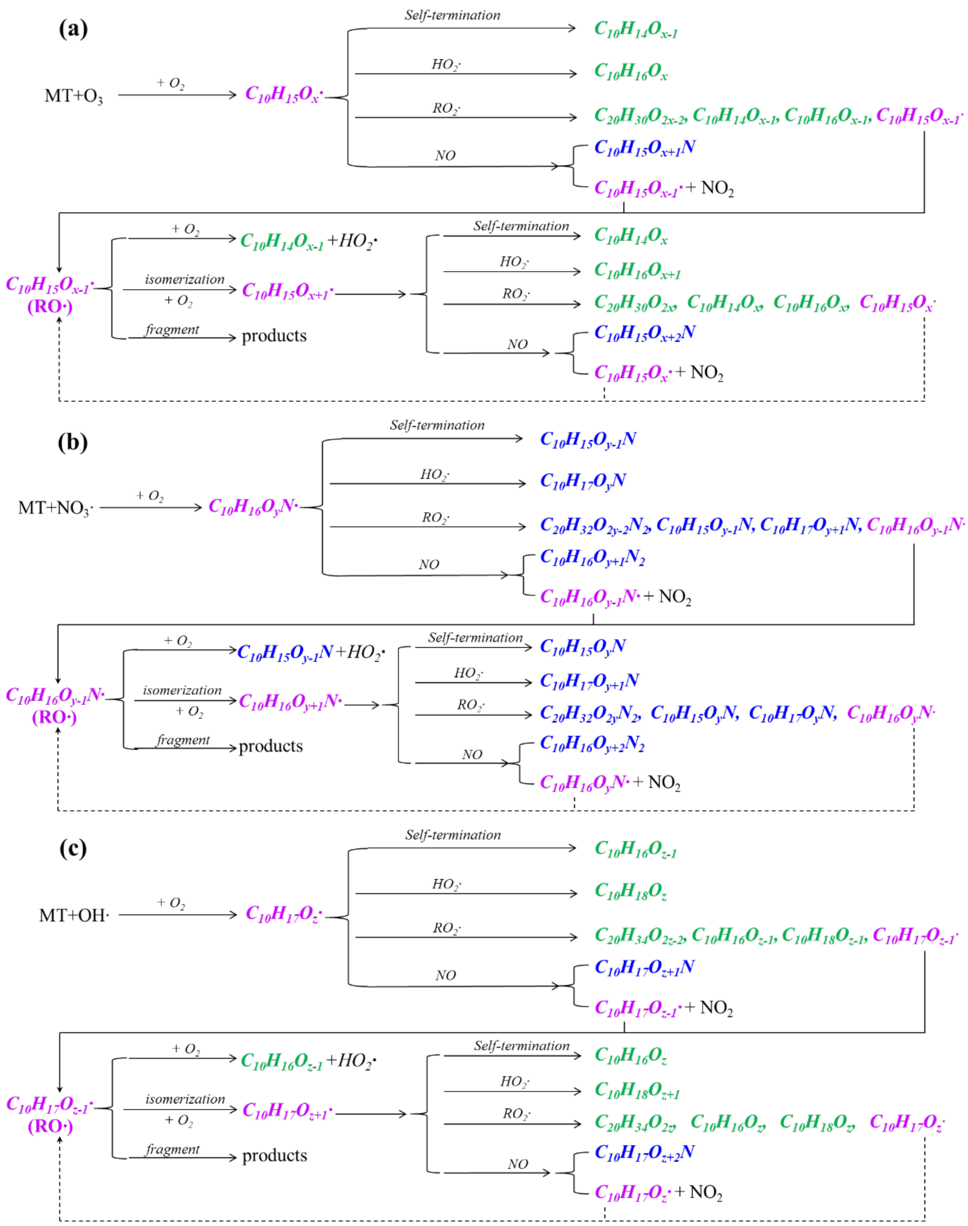

Supplementary Figure 5 Diagram showing the formation pathways of highly oxygenated organic molecules (HOMs) from (a) O_3 oxidation, (b) NO_3 oxidation, and (c) OH oxidation in the presence of NO_x. Close shell CHO molecules are highlighted in green, peroxy radical in purple and CHON in blue. x, y and z are the oxygen number with even numbers of x and odd numbers of y and z. Molecules with the same formula can be formed via different pathways. E.g., $C_{10}H_{14}O_{x-1}$ and $C_{10}H_{15}O_x$· can be formed from both NO_x-free and NO_x-involved pathways; $C_{10}H_{15}O_{y-1}N$ from NO_3 channel and $C_{10}H_{15}O_{x+2}N$ from NO channel can be the same molecular formula. Noting that the oxidation of monoterpenes by OH and NO_3 is assumed to be an addition reaction rather than a H-abstraction reaction.

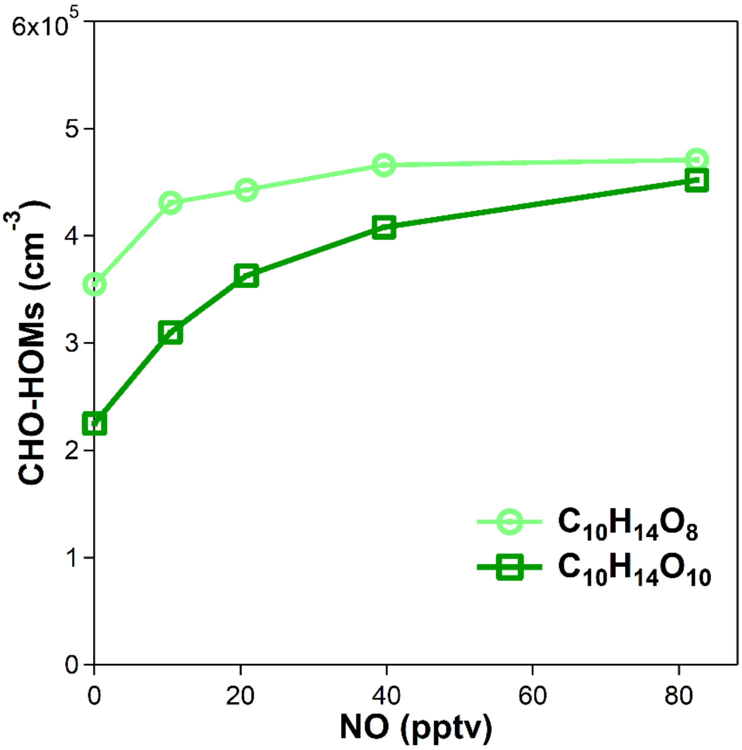

Supplementary Figure 6 Major highly oxygenated organic molecules (HOMs) without nitrogen atoms (CHO-HOM molecules) produced from RO auto-oxidation and their dependences on NO concentration in varying NO/NO_2 experiment run with 1200 ppt monoterpene

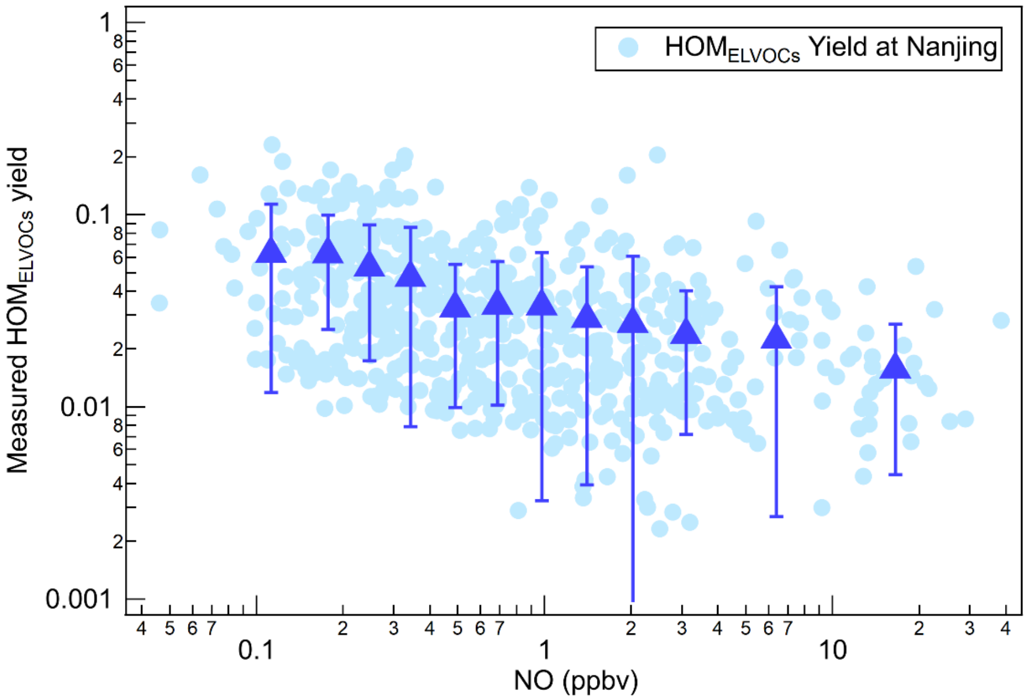

Supplementary Figure 7 Dependence of the yields of extremely low volatility fraction of highly oxygenated organic molecules (HOM$_{ELVOCs}$) on NO in polluted east China. The HOM$_{ELVOCs}$ yield was calculated via equation (5), excluding the possible contribution of multi-stage oxidation. The light blue data points are the half-hour average data points. The dark blue triangles represent the mean values of HOM$_{ELVOCs}$ yield in the bins of NO concentrations of <0.15, 0.15-0.2, 0.2-0.3, 0.3-0.4, 0.4-0.6, 0.6-0.8, 0.8-1.2, 1.2-1.6, 1.6-2.5, 2.5-4, 4-10 and >10 ppbv. Error bars are the standard deviations. The measurement campaign was conducted from 2 August to 6 September 2019 at SORPES station in Nanjing, China.

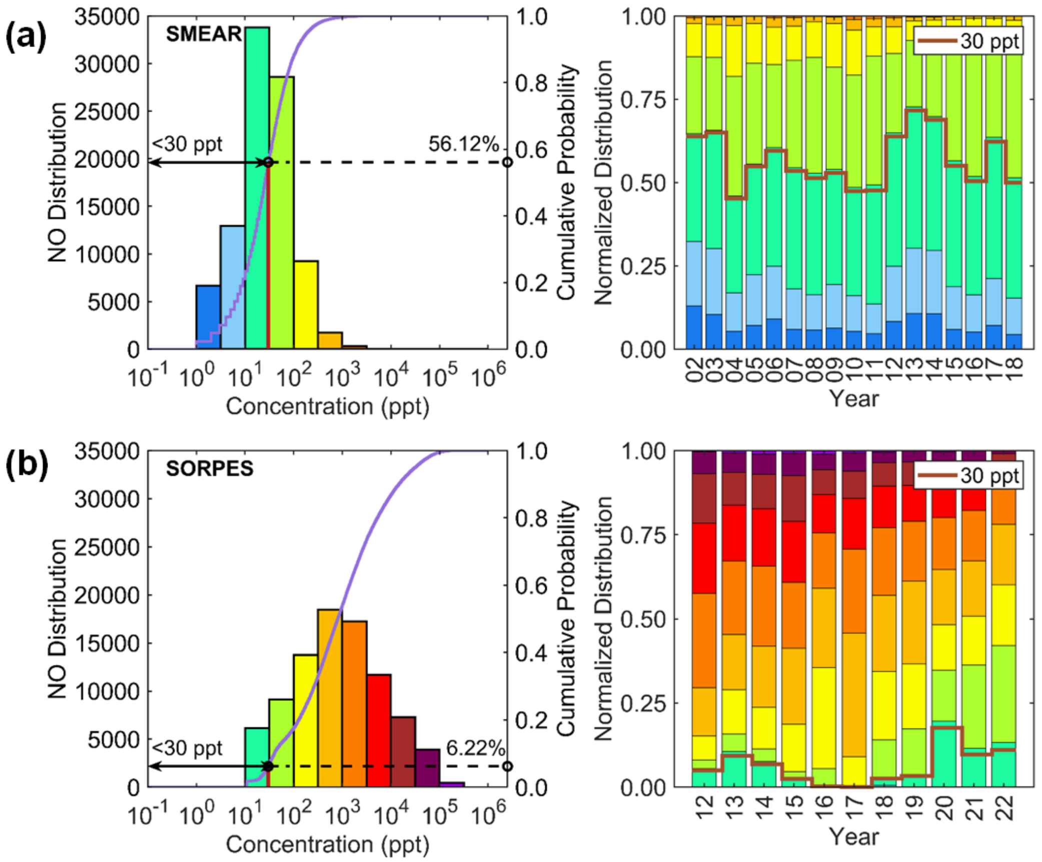

Supplementary Figure 8 Distribution of NO concentration at (a) SEMAR II station in a boreal forest from 2002 to 2018, and (b) SORPES station in polluted east China from 2012 to 2022.

Supplementary Table 1 Experimental setting of involved runs in this study. In the pure NO_2 experiment run, we added NO_2 at different concentrations; in constant NO/NO_2 experiment runs, we added NO at different concentrations, and due to the O_3, the NO converted to NO_2, leading to a NO/NO_2 ratio of ~0.7%; in varying NO/NO_2 experiment run, we added around 1 ppb NO_2, and adjusted NO concentrations from 0 to 82 pptv by playing with the UV light intensity. In pure NO_2 and constant NO/NO_2 runs, NO_x was introduced in approximate concentrations of 0.7, 1.9, and 4.6 ppbv, representing very clean to slightly polluted environments.

	**Runs**	**NO_x (ppb)**	**NO (ppt)**	**Monoterpene (ppt)**	**Ozone (ppb)**
a	Pure NO_2	0, 0.7, 1.9, 4.6	0	1200	40
b	Constant NO/NO_2	0, 0.7, 1.9, 4.6	0, 5, 12, 28	1200	40~36
c	Constant NO/NO_2	0, 0.7, 1.9, 4.6	0, 5, 12, 28	300	40~36
d	Varying NO/NO_2	~1ppb	0 ~ 82	1200	40

Supplementary Table 2 Division of different NO_x regimes. For the classical division, autoxidation is not considered; NO_x regimes are simply differentiated into "Low NO_x" and "High NO_x" to denote RO_2 reactions with NO_x being negligible or dominant. In most previous chamber studies, "High NO_x" usually represents the concentration of NO_x much higher than the real atmosphere, while "Low NO_x" represents the background NO_x concentration in the chamber without any injection. In this study, we demonstrated that zero NO_x is significantly different from "Low NO_x" and NO is the key species affecting the fate of RO_2. Therefore, we proposed a new division of NO_x regimes as "Non-NOx", "Low NO", and "High NO".

**Classical division**	
**"Low-NO_x"**	RO_2 reactions with NO_x negligible
**"High-NO_x"**	RO_2 reactions with NO_x dominate
Notes	*Autoxidation not considered*
**Proposed division**	
**"Zero-NO_x"**	RO_2 reactions with NO_x negligible
**"Low-NO"**	Some RO_2 reactions with NO, autoxidation promoted
**"High-NO"**	RO_2 reactions with NO dominate, RO_2 autoxidation hampered, RO isomerization can still lead to HOM

Supplementary Table 3 The main yield, branch ratio, and autoxidation rate constants deployed in the ADCHAM model in this study

Paramters in PRAM	Values	Description of parameters
yO3_APIN_PRAM1	0.0375	The yield of $C_{10}H_{15}O_4\cdot$-isomer 1 that can initiate the autoxidation from APINOOA*
yO3_APIN_PRAM2	0.0375	The yield of $C_{10}H_{15}O_4\cdot$-isomer 2 that can initiate the autoxidation from APINOOA
yO3_APIN_PRAM3	0.0225	The yield of $C_{10}H_{15}O_4\cdot$-isomer 3 that can initiate the autoxidation from APINOOA
yO3_APIN_PRAM4	0.0375	The yield of $C_{10}H_{15}O_4\cdot$-isomer 4 that can initiate the autoxidation from APINOOA
yO3_APIN_PRAM5	0.0075	The yield of $C_{10}H_{15}O_4\cdot$-isomer 5 that can initiate the autoxidation from APINOOA
yO3_APIN_PRAM6	0.0075	The yield of $C_{10}H_{15}O_4\cdot$-isomer 5 that can initiate the autoxidation from APINOOA
yOH_APIN_PRAM1	0	The yield of $C_{10}H_{17}O_3\cdot$-isomer 1 from α-pinene + OH reaction
yOH_APIN_PRAM2	0.0075	The yield of $C_{10}H_{17}O_3\cdot$-isomer 2 from α-pinene + OH reaction
yOH_APIN_PRAM3	0.0037	The yield of $C_{10}H_{17}O_3\cdot$-isomer 3 from α-pinene + OH reaction
yNO3_APIN_PRAM1	0	The yield of $C_{10}H_{16}NO_5\cdot$-isomer 1 from α-pinene + NO_3 reaction
yNO3_APIN_PRAM2	0	The yield of $C_{10}H_{16}NO_5\cdot$-isomer 2 from α-pinene + NO_3 reaction
yNO3_APIN_PRAM3	0	The yield of $C_{10}H_{16}NO_5\cdot$-isomer 3 from α-pinene + NO_3 reaction
yO3_CAR_PRAM1	0.0375	The yield of $C_{10}H_{15}O_4\cdot$-isomer 1 that can initiate the autoxidation from APINOOA
yO3_CAR_PRAM2	0.0375	The yield of $C_{10}H_{15}O_4\cdot$-isomer 2 that can initiate the autoxidation from APINOOA
yO3_CAR_PRAM3	0.0225	The yield of $C_{10}H_{15}O_4\cdot$-isomer 3 that can initiate the autoxidation from APINOOA
yO3_CAR_PRAM4	0.0375	The yield of $C_{10}H_{15}O_4\cdot$-isomer 4 that can initiate the autoxidation from APINOOA
yO3_CAR_PRAM5	0.0075	The yield of $C_{10}H_{15}O_4\cdot$-isomer 5 that can initiate the autoxidation from APINOOA
yO3_CAR_PRAM6	0.0075	The yield of $C_{10}H_{15}O_4\cdot$-isomer 6 that can initiate the autoxidation from APINOOA
yOH_CAR_PRAM1	0	The yield of $C_{10}H_{17}O_3\cdot$-isomer 1 from δ-3-carene + OH reaction
yOH_CAR_PRAM2	0	The yield of $C_{10}H_{17}O_3\cdot$-isomer 2 from δ-3-carene + OH reaction
yOH_CAR_PRAM3	0	The yield of $C_{10}H_{17}O_3\cdot$-isomer 3 from δ-3-carene + OH reaction
yNO3_CAR_PRAM1	0.045	The yield of $C_{10}H_{16}NO_5\cdot$-isomer 1 from α-pinene + NO_3 reaction
yNO3_CAR_PRAM2	0.165	The yield of $C_{10}H_{16}NO_5\cdot$-isomer 2 from α-pinene + NO_3 reaction
yNO3_CAR_PRAM3	0.015	The yield of $C_{10}H_{16}NO_5\cdot$-isomer 3 from α-pinene + NO_3 reaction
func_RO2_NO_O4	0.8	Branch ratio of RO, which can isomerize via H-abstraction, from $C_{10}H_{15}O_4\cdot$+ NO reaction
func_RO2_NO_O5	0.6	Branch ratio of RO, which can isomerize via H-abstraction, from $C_{10}H_{15}O_5\cdot$+ NO reaction
func_RO2_NO_O6	0.5	Branch ratio of RO, which can isomerize via H-abstraction, from $C_{10}H_{15}O_6\cdot$+ NO reaction
func_RO2_NO_O7	0.4	Branch ratio of RO, which can isomerize via H-abstraction, from $C_{10}H_{15}O_7\cdot$+ NO reaction
func_RO2_NO_O8	0.2	Branch ratio of RO, which can isomerize via H-abstraction, from $C_{10}H_{15}O_8\cdot$+ NO reaction
func_RO2_NO_O9	0	Branch ratio of RO, which can isomerize via H-abstraction, from $C_{10}H_{15}O_9\cdot$+ NO reaction
func_RO2_NO_O10	0	Branch ratio of RO, which can isomerize via H-abstraction,

		from $C_{10}H_{15}O_{10}\cdot$+ NO reaction
func_RO2_NO_O11	0	Branch ratio of RO, which can isomerize via H-abstraction, from $C_{10}H_{15}O_{11}\cdot$+ NO reaction
func_RO2_NO_O12	0	Branch ratio of RO, which can isomerize via H-abstraction, from $C_{10}H_{15}O_{12}\cdot$+ NO reaction
func_RO2_NO_O13	0	Branch ratio of RO, which can isomerize via H-abstraction, from $C_{10}H_{15}O_{13}\cdot$+ NO reaction
func_OH_RO2_NO_O3	0.9	Branch ratio of RO, which can isomerize via H-abstraction, from $C_{10}H_{17}O_{3}\cdot$+ NO reaction
func_OH_RO2_NO_O4	0.9	Branch ratio of RO, which can isomerize via H-abstraction, from $C_{10}H_{17}O_{4}\cdot$+ NO reaction
func_OH_RO2_NO_O5	0.9	Branch ratio of RO, which can isomerize via H-abstraction, from $C_{10}H_{17}O_{5}\cdot$+ NO reaction
func_OH_RO2_NO_O6	0.9	Branch ratio of RO, which can isomerize via H-abstraction, from $C_{10}H_{17}O_{6}\cdot$+ NO reaction
func_OH_RO2_NO_O7	0.8	Branch ratio of RO, which can isomerize via H-abstraction, from $C_{10}H_{17}O_{7}\cdot$+ NO reaction
func_OH_RO2_NO_O8	0.7	Branch ratio of RO, which can isomerize via H-abstraction, from $C_{10}H_{17}O_{8}\cdot$+ NO reaction
func_OH_RO2_NO_O9	0	Branch ratio of RO, which can isomerize via H-abstraction, from $C_{10}H_{17}O_{9}\cdot$+ NO reaction
func_NO3_RO2_NO_O5	1	Branch ratio of RO, which can isomerize via H-abstraction, from $C_{10}H_{16}NO_{5}\cdot$+ NO reaction
func_NO3_RO2_NO_O6	0.6	Branch ratio of RO, which can isomerize via H-abstraction, from $C_{10}H_{16}NO_{6}\cdot$+ NO reaction
func_NO3_RO2_NO_O7	0.4	Branch ratio of RO, which can isomerize via H-abstraction, from $C_{10}H_{16}NO_{7}\cdot$+ NO reaction
func_NO3_RO2_NO_O8	0.3	Branch ratio of RO, which can isomerize via H-abstraction, from $C_{10}H_{16}NO_{8}\cdot$+ NO reaction
func_NO3_RO2_NO_O9	0.2	Branch ratio of RO, which can isomerize via H-abstraction, from $C_{10}H_{16}NO_{9}\cdot$+ NO reaction
func_NO3_RO2_NO_O10	0	Branch ratio of RO, which can isomerize via H-abstraction, from $C_{10}H_{16}NO_{10}\cdot$+ NO reaction
func_NO3_RO2_NO_O11	0	Branch ratio of RO, which can isomerize via H-abstraction, from $C_{10}H_{16}NO_{11}\cdot$+ NO reaction
func_NO3_RO2_NO_O12	0	Branch ratio of RO, which can isomerize via H-abstraction, from $C_{10}H_{16}NO_{12}\cdot$+ NO reaction
func_NO3_RO2_NO_O13	0	Branch ratio of RO, which can isomerize via H-abstraction, from $C_{10}H_{16}NO_{13}\cdot$+ NO reaction
kautoox_O4	1D17*EXP(-1.2077D4/Temp)	Autoxidation reaction rate constant of $C_{10}H_{15}O_{4}\cdot$**
kautoox_O5	5D16*EXP(-1.2077D4/Temp)	Autoxidation reaction rate constant of $C_{10}H_{15}O_{5}\cdot$
kautoox_O6	1D17*EXP(-1.2077D4/Temp)	Autoxidation reaction rate constant of $C_{10}H_{15}O_{6}\cdot$
kautoox_O7	1D17*EXP(-1.2077D4/Temp)	Autoxidation reaction rate constant of $C_{10}H_{15}O_{7}\cdot$
kautoox_O8	1D19*EXP(-1.2077D4/Temp)	Autoxidation reaction rate constant of $C_{10}H_{15}O_{8}\cdot$
kautoox_O9	1D19*EXP(-1.2077D4/Temp)	Autoxidation reaction rate constant of $C_{10}H_{15}O_{9}\cdot$
kautoox_O10	2D17*EXP(-1.2077D4/Temp)	Autoxidation reaction rate constant of $C_{10}H_{15}O_{10}\cdot$
kautoox_O11	5D16*EXP(-1.2077D4/Temp)	Autoxidation reaction rate constant of $C_{10}H_{15}O_{11}\cdot$
kautoox_O12	1D16*EXP(-1.2077D4/Temp)	Autoxidation reaction rate constant of $C_{10}H_{15}O_{12}\cdot$
kautoox_OH_O3	3D17*EXP(-1.2077D4/Temp)	Autoxidation reaction rate constant of $C_{10}H_{17}O_{3}\cdot$
kautoox_OH_O4	3D17*EXP(-1.2077D4/Temp)	Autoxidation reaction rate constant of $C_{10}H_{17}O_{4}\cdot$
kautoox_OH_O5	2D18*EXP(-1.2077D4/Temp)	Autoxidation reaction rate constant of $C_{10}H_{17}O_{5}\cdot$
kautoox_OH_O6	1D17*EXP(-1.2077D4/Temp)	Autoxidation reaction rate constant of $C_{10}H_{17}O_{6}\cdot$
kautoox_OH_O7	1D17*EXP(-1.2077D4/Temp)	Autoxidation reaction rate constant of $C_{10}H_{17}O_{7}\cdot$
kautoox_NO3_O5	0*EXP(-1.2077D4/Temp)	Autoxidation reaction rate constant of $C_{10}H_{16}NO_{5}\cdot$
kautoox_NO3_O6	5D16*EXP(-1.2077D4/Temp)	Autoxidation reaction rate constant of $C_{10}H_{16}NO_{6}\cdot$
kautoox_NO3_O7	1D18*EXP(-1.2077D4/Temp)	Autoxidation reaction rate constant of $C_{10}H_{16}NO_{7}\cdot$
kautoox_NO3_O8	1D17*EXP(-1.2077D4/Temp)	Autoxidation reaction rate constant of $C_{10}H_{16}NO_{8}\cdot$
kautoox_NO3_O9	1D18*EXP(-1.2077D4/Temp)	Autoxidation reaction rate constant of $C_{10}H_{16}NO_{9}\cdot$
kautoox_NO3_O10	1D17*EXP(-1.2077D4/Temp)	Autoxidation reaction rate constant of $C_{10}H_{16}NO_{10}\cdot$

* APINOOA is criegee intermediate isomer 1 from ozonolysis of α-pinene.

** The units of all reaction rate constants are cm^3 $molec^{-1}$ s^{-1}; for the complete model setup, please refer to Roldin et al., 2019[33].
